# Ferroptosis-Related Gene Model to Predict Overall Survival of Ovarian Carcinoma

**DOI:** 10.1155/2021/6687391

**Published:** 2021-01-13

**Authors:** Liuqing Yang, Saisai Tian, Yun Chen, Chenyun Miao, Ying Zhao, Ruye Wang, Qin Zhang

**Affiliations:** ^1^Guangxing Hospital Affiliated to Zhejiang Chinese Medical University, Hangzhou, Zhejiang 310007, China; ^2^Department of Phytochemistry, School of Pharmacy, The Second Military Medical University, Shanghai 200433, China; ^3^Zhejiang Chinese Medical University, Hangzhou, Zhejiang 310053, China

## Abstract

**Background:**

Ovarian cancer (OC) is the eighth most common cause of cancer death and the second cause of gynecologic cancer death in women around the world. Ferroptosis, an iron-dependent regulated cell death, plays a vital role in the development of many cancers. Applying expression of ferroptosis-related gene to forecast the cancer progression is helpful for cancer treatment. However, the relationship between ferroptosis-related genes and OC patient prognosis is still vastly unknown, making it still a challenge for developing ferroptosis therapy for OC.

**Methods:**

The Cancer Genome Atlas (TCGA) data of OC were obtained and the datasets were randomly divided into training and test datasets. A novel ferroptosis-related gene signature associated with overall survival (OS) was constructed according to the training cohort. The test dataset and ICGC dataset were used to validate this signature.

**Results:**

We constructed a model containing nine ferroptosis-related genes, namely, *LPCAT3*, *ACSL3*, *CRYAB*, *PTGS2*, *ALOX12*, *HSBP1*, *SLC1A5*, *SLC7A11*, and *ZEB1*, and predicted the OS of OC in TCGA. At a suitable cutoff, patients were divided into low risk and high risk groups. The OS curves of the two groups of patients had significant differences, and the time-dependent receiver operating characteristics (ROCs) were as high as 0.664, respectively. Then, the test dataset and the ICGC dataset were used to evaluate our model, and the ROCs of test dataset were 0.667 and 0.777, respectively. In addition, functional analysis and correlation analysis showed that immune-related pathways were significantly enriched. Meanwhile, we also integrated with other clinical factors and we found the synthesized clinical factors and ferroptosis-related gene signature improved prognostic accuracy relative to the ferroptosis-related gene signature alone.

**Conclusion:**

The ferroptosis-related gene signature could predict the OS of OC patients and improve therapeutic decision-making.

## 1. Introduction

Ovarian cancer (OC) is the eighth most common cause of cancer death and the second cause of gynecology cancer death in women around the world [1]. Among all types of OCs, epithelial OC (EOC) accounts for over 95% of all ovarian malignancies [2, 3]. OC is heterogeneous and the etiology remains complicated and uncertain [4, 5]. Risk factors include inherited risk, obesity, age, perineal talc use, etc. [3, 6]. The prognosis of OC relies on the stage and early prevention. Over the past years, improved screening, surgery, and treatment methods have contributed largely to the increase of survival. However, survival rates for OC have changed modestly for decades, even in developed countries such as America and Canada [3]. Approximately 70% of OCs are diagnosed at an advanced stage and have a relatively low 5-year survival rate of 30% [7]. Uncertain etiologic factors and low survival rate of OC make the finding of novel therapeutic strategies and models urgent.

Ferroptosis, first coined in 2012, is an iron-dependent and reactive oxygen species (ROS) reliant form of regulated cell death (RCD) [8, 9]. Emerging evidence shows that ferroptosis acts like a nexus between metabolism, redox biology, and human health [10]. In recent years, ferroptosis has been exhibiting huge potential of triggering cancer cell death by regulating the mechanism of iron metabolism, amino acid and glutathione metabolism, and ROS metabolism, particularly for eradicating aggressive malignancies that are resistant to conventional therapies [10]. Lately, ferroptosis has been reported to play a vital role in the progression of OC and genes like stearoyl-CoA desaturase 1 could protect OC cells from ferroptosis cell death [11, 12]. TAZ-ANGPTL4-NOX2 axis regulates ferroptosis cell death and chemoresistance in EOC [13]. On the other hand, ferroptosis-regulator gene glutathione peroxidase 4 (*GPX4*) is highly associated with tumorigenesis and progression [14, 15]. Therefore, ferroptosis can be a potential and powerful target for cancer therapy. However, the relationship between ferroptosis-related genes and OC patient prognosis is still vastly unknown, making it still a challenge for developing ferroptosis therapy for OC.

In this paper, we downloaded OC patient samples from publicity datasets TCGA and ICGC. After preprocessing the data, we constructed a prognostic model composed of nine ferroptosis-related genes in TCGA training set and validated it in TCGA test dataset and ICGC dataset. Further, we conducted functional annotation to discover the possible mechanisms. Finally, restricted median survival (RMS) analysis was applied to combine and evaluate the clinical information and the constructed model. The results showed that the combination had stronger power than the risk model only.

## 2. Material and Methods

### 2.1. Data Collection and Preprocessing

**Figure 1:**
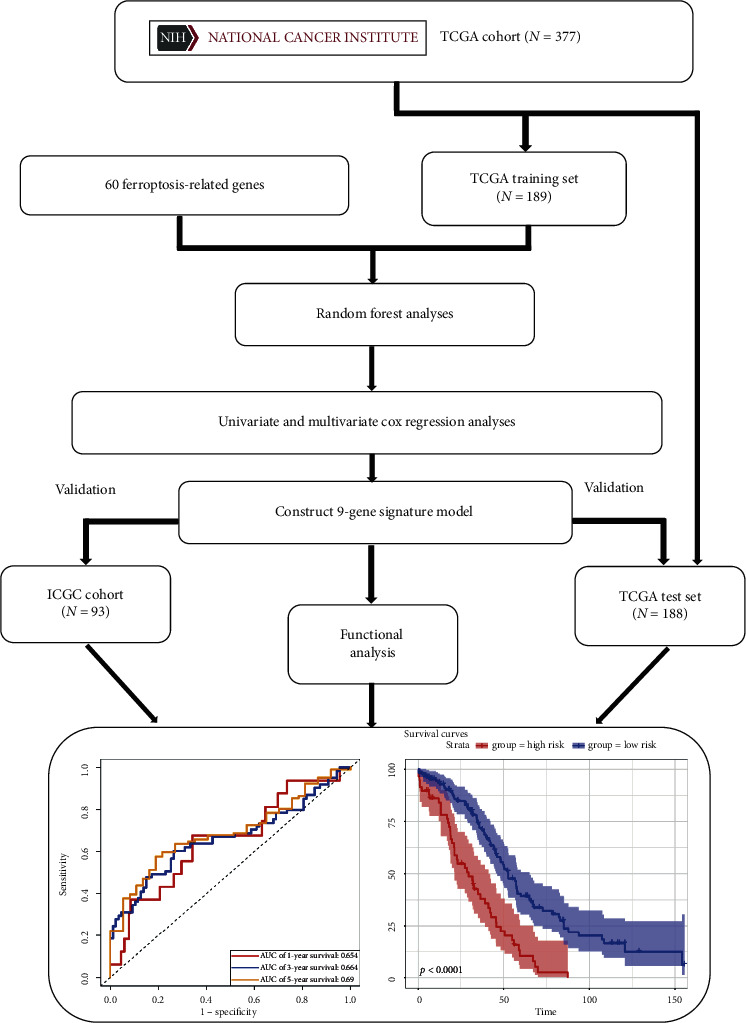
Flowchart of data collection and analysis.

All datasets used in this study were publicly available and the workflow of this work is shown in Figure 1. The count data of OC were obtained from The Cancer Genome Atlas (TCGA) (https://tcga-data.nci.nih.gov/tcga/). A total of 377 OC patient samples with corresponding clinical information were available in TCGA. The detail information of clinical data about 377 samples is shown in Table 1. For raw count data, we first transformed the Ensembl IDs to gene symbols and protein-coding gene was selected for this research. Then, we computed the transcripts per kilobase million (TPM) values, which were more comparable between samples. Meanwhile, the expression data and clinical information of OC were downloaded from International Cancer Genome Consortium (ICGC) resource (https://dcc.icgc.org/). Finally, according to the previous literatures [16–19], 60 ferroptosis-related genes were collected and listed in Supplementary Table S1.

### 2.2. Construction of Risk Model and Ferroptosis-Related Feature Signature

After data preprocessing, 50% of samples were randomly divided into training set (containing 189 OC samples) and another 50% were allocated as validation set (containing 188 OC samples). First, ferroptosis-related genes with prognostic values were identified by univariate cox analysis of overall survival (OS) in the training set selected in TCGA data. The coxph function in the survival R package was used, and *p* < 0.15 was selected as the threshold. Finally, 15 ferroptosis-related genes were screened (Supplementary Table S2). Further, feature selection was conducted by the randomForestSRC R package. The random forest algorithm was used for ranking the importance of prognostic genes. Only genes with variable relative importance >0.4 were identified as the final signature. Then, we performed multivariate cox analysis on the final signature obtained from the random forest algorithm. Finally, using a linear combination in the training datasets, a formula for the risk score was established. The hazards model was constructed as follows:

(1) $$\begin{array}{c} \text{RiskScore}=\sum_{i=1}^{N}\left(\exp\;*\text{coef}\right), \end{array}$$

where *N* is the number of gene, exp is the expression value of gene, and coef is the coefficient of gene in the multivariate cox analysis.

### 2.3. The Robustness Verification of the Gene Signature in Internal and External Datasets

Risk score and overall survival (OS) analysis were performed using the coxph function in the survival R package. The sensitivity and specificity of the model were assessed by the receiver operating characteristic (ROC) curve, drawn by using the timeROC R package, and were used for analyzing the prognosis prediction of 1 year, 3 years, and 5 years [20]. Then, to verify the stability of the model obtained, the performance of the model was evaluated in TCGA test dataset and ICGC cohort.

### 2.4. Estimation of the Abundance of Immune Cell Populations

In this study, 24 tumor-infiltrating immune cells (TIICs) from the literature [21] that included two categories of adaptive immunity and innate immunity were used to calculate the infiltration level of specific immune cell using Single-Sample Gene Set Enrichment Analysis (ssGSEA) algorithm. In brief, ssGSEA applied gene signatures expressed by immune cell populations to individual cancer samples and we used ssGSEA algorithm to estimate the infiltration levels of 24 kinds of TIICs in OC samples. In our research, the ssGSEA algorithm was implemented in the gsva R package.

### 2.5. Functional Annotation Analysis

In the training dataset, patients with OC were divided into two groups, including high risk and low risk groups, according to the optimal cutoff value. To identify the potentially altered pathways between high risk and low risk groups, the Kyoto Encyclopedia of Genes and Genomes (KEGG) enrichment analysis and Gene Ontology (GO) analysis were applied for gene set annotation, and GSEA algorithm was applied to identify the key pathways and biological process by using the R package “clusterProfiler.”

### 2.6. Statistical Analysis

Statistical analysis was performed using the R software (3.6.2 version, https://cran.r-project.org/). Student's *t*-test was used to evaluate the difference between different groups. Chi-squared test was used to compare the differences in different proportions. The ssGSEA scores between two groups were compared by Mann–Whitney test with *p* values (adjusted by the BH method). The Kaplan–Meier method was applied to perform OS analysis. The differences of OS between two groups were assessed by two-sided log rank tests. *p* value <0.05 was regarded statistically significant.

## 3. Results

### 3.1. Identification of Nine Ferroptosis-Related Genes

**Figure 2:**
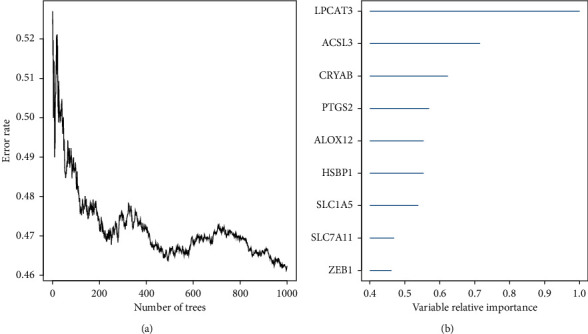
(a) Error rate for the data as a function of the classification tree. (b) Out of bag importance values for the nine ferroptosis-related genes.

In this paper, 60 ferroptosis-related genes were processed by randomForestSRC R package for gene feature selection. Ferroptosis-related genes with relative importance >0.4 were considered as the final signature. The relationship between the error rate and the number of classification trees is shown in Figure 2(a). After ranking these genes according to the importance of out of bag, 9 top ferroptosis-related genes are shown in Figure 2(b). These genes are *LPCAT3*, *ACSL3*, *CRYAB*, *PTGS2*, *ALOX12*, *HSBP1*, *SLC1A5*, *SLC7A11*, and *ZEB1*.

### 3.2. Construction Genes Weighted by Their Coefficients from a Ferroptosis-Related Prognosis Model in TCGA Cohort

By linearly combining the nine ferroptosis-related genes weighted by their coefficients from multivariate cox analysis, a hazard model was constructed as a formula:

Riskscore = (0.1339*∗*E_*ZEB1*_) + (0.3175*∗*E_*SLC7A11*_) + (0.1769*∗*E_*SLC1A5*_) + (0.0923*∗*E_*HSBP1*_) + (0.2194*∗*E_*ALOX12*_) + (0.0024*∗*E_*PTGS2*_) + (0.1861*∗*E_*CRYAB*_) + (0.4275*∗*E_*ACSL3*_) + (0.2694*∗*E_*LPCAT3*_).E_*ZEB1*_ is the expression value of gene *ZEB1*. The rest are similar to gene *ZEB1*.

**Figure 3:**
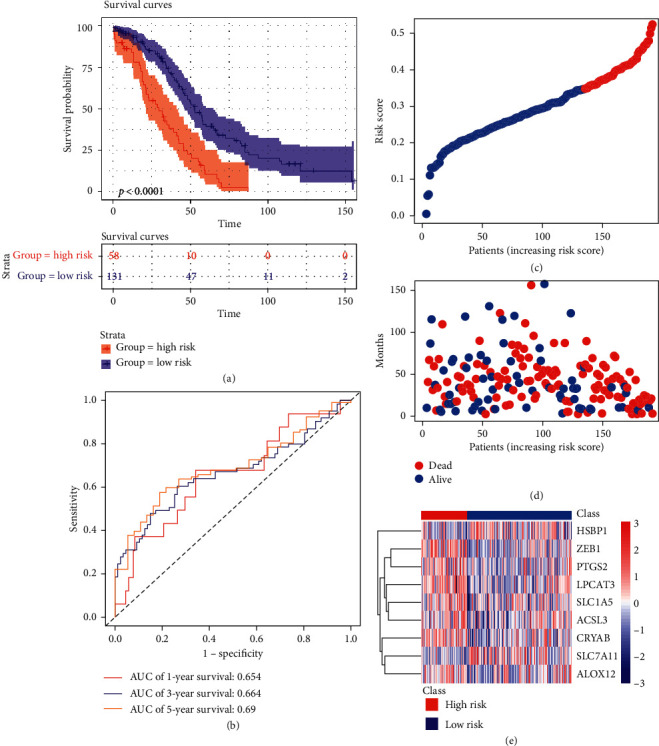
Prognosis analysis of nine ferroptosis-related genes in TCGA training set. (a) Kaplan–Meier curves for the OS of patients in the two groups. (b) ROC curves. (c) The detailed risk scores of patients. (d) Survival status of patients. (e) Heatmap of the nine ferroptosis-related genes between high and low risk group.

The risk score of each sample was calculated using the above method. The patients in TCGA training cohort were divided into high risk group (*n* = 58) and low risk group (*n* = 131) according to the optimal cutoff value determined by survminer package in R. As the Kaplan–Meier curves show in Figure 3(a), people in high risk group have a higher probability of death than those in the low risk group (*p* < 0.0001). The ROC analysis is shown in Figure 3(b) and the ROC curves reach 0.654 at 1 year, 0.664 at 3 years, and 0.69 at 5 years. And the detailed risk score, survival information, and ferroptosis-related genes' expression are displayed (Figures 3(c)–3(e)).

### 3.3. Validation of the Nine Ferroptosis Genes' Signature Using the Test Dataset

**Figure 4:**
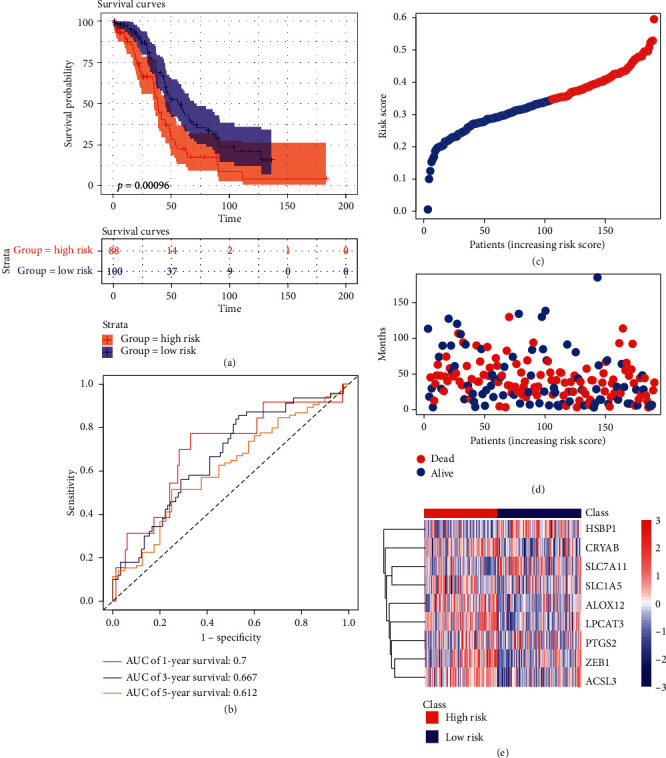
Validation of the nine ferroptosis-related gene model in TCGA test set. (a) Kaplan–Meier curves for the OS of patients in the two groups. (b) ROC curves. (c) The detailed risk scores of patients. (d) Survival status of patients in TCGA test set. (e) Heatmap of the nine ferroptosis-related genes between high and low risk group.

The robustness of the model was examined in the test dataset from TCGA cohort (*n* = 188), including 88 samples in high risk group and 100 samples in low risk group according to the same risk formula. Patients in higher risk group had poorer survival time than those in low risk group, consistent with the former results (Figure 4(a)). The AUC of time-dependent ROC in 1 year, 3 years, and 5 years is 0.7, 0.667, and 0.612, respectively (Figure 4(b)). The detailed risk score, survival information, and ferroptosis-related genes' expression also are displayed (Figures 4(c)–4(e)).

### 3.4. Validation of the Nine Ferroptosis-Related Genes' Signature in ICGC Cohort

**Figure 5:**
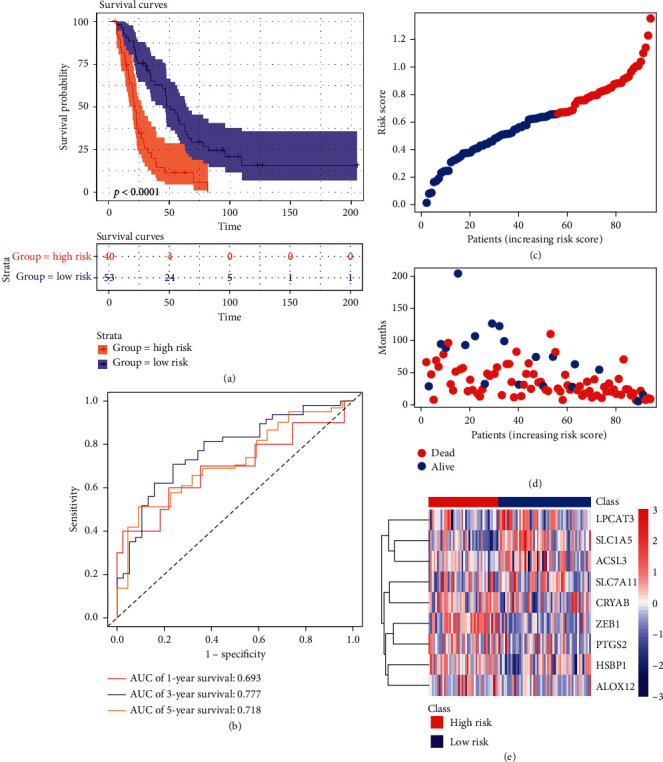
Validation of the nine ferroptosis-related genes signature in ICGC cohort. (a) Survival analysis of patients in ICGC cohort. (b) AUC of ROC. (c) The detailed risk scores of patients. (d) The survival status of patients. (e) Heatmap of the nine ferroptosis-related genes.

To further test the robustness of the constructed model, patients (*n* = 93) from the ICGC cohort were categorized into high risk group (40 samples) and low risk group (53 samples) according to the same risk formula above. The survival curves show the patients in high risk group had lower survival probability than patients in low risk group (*p* < 0.0001) (Figure 5(a)). The AUC of the model was 0.693 at 1 year, 0.777 at 3 years, and 0.718 at 5 years (Figure 5(b)). The detailed risk scores, survival information, and nine ferroptosis-related genes' expression in ICGC cohort are shown (Figures 5(c)–5(e)).

### 3.5. Independent Prognostic Factor of the Gene Signature

We carried out univariate and multivariate cox analysis to determine whether the gene signature was an independent prognostic predictor. Applying univariate cox regression analysis, we found the risk score was significantly associated with OS in the training dataset, test dataset, and the ICGC cohort (HR = 2.657, 95% CI = 1.823–3.872, *p* < 0.001; HR = 1.887, 95% CI = 1.287–2.768, *p* < 0.001; HR = 3.115, 95% CI = 1.914–5.069, *p* < 0.001, respectively) (Table 2). After correction for other confounding factors by the multivariate cox regression analysis, the risk score still proved to be an independent predictor for OS (HR = 1.767, 95% CI = 1.155–2.704, *p*=0.009; HR = 1.944, 95% CI = 1.271–2.973, *p*=0.002; HR = 3.06, 95% CI = 1.865–5.021, *p* < 0.001, respectively). In addition, the ferroptosis-related gene model also was assessed on the clinical factors, including age, stage, and grade tumor status of the tumor, and the Kaplan–Meier analyses revealed that patients in the high risk of death group had significantly shorter OS compared with patients in the low risk of death group in the training dataset, test dataset, and ICGC dataset (*p* < 0.05) (Figure S1).

### 3.6. The Relationship between Risk Scores and Immune Status

**Figure 6:**
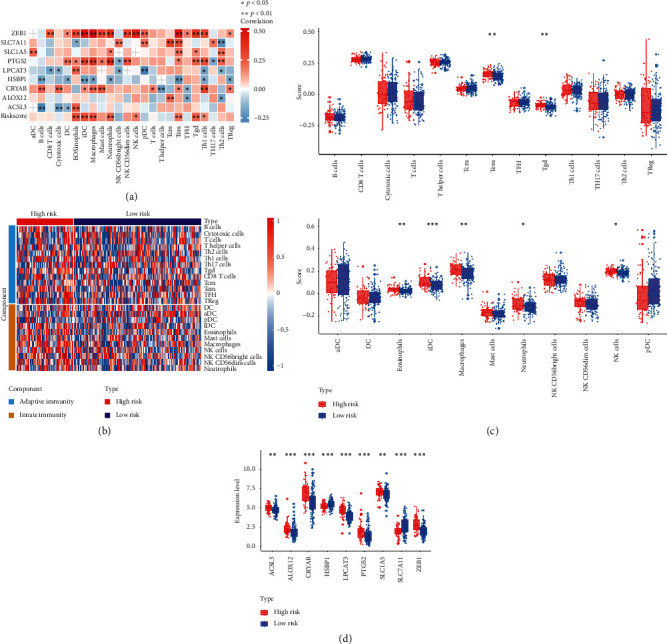
Comparison of the ssGSEA scores between different risk groups in the TCGA training set. (a) The correlation between the nine ferroptosis-related genes and risk score and different immune cells. (b) Heatmap of the immune cell infiltration between different groups. (c) Detailed risk scores and comparison in high risk group and low risk group. (d) The expression level and comparison of nine ferroptosis-related genes in high risk group and low risk group. The meaning of the statistical difference is as follows: ^*∗*^*p* < 0.05, ^*∗∗*^*p* < 0.01, and ^*∗∗∗*^*p* < 0.001.

Considering ferroptosis was strongly associated with immune status, we further explored the correlations between risk scores and immune status using the ssGSEA method. The different subpopulations of immune cells were divided into adaptive immunity cells and innate immunity cells. First, the correlation analysis between the nine ferroptosis-related genes and risk scores and the abundance of immune cells are shown in Figure 6(a). The results showed that the risk scores and the nine ferroptosis-related genes meet strong correlations with most of the immune cells, such as eosinophils, iDC, macrophages, neutrophils, NK cells, Tem, Tgd, and Th1 cells, suggesting strong connections between the nine ferroptosis-related genes and immune status. Then, heatmap and the boxplot of ssGSEA scores of adaptive immunity cells and innate immunity cells between high risk patients and low risk patients in TCGA training cohort are shown in Figures 6(b) and 6(c). In addition, the nine ferroptosis-related genes in high risk group and low risk group were compared and the results are shown in Figure 6(d). The expression level of nine ferroptosis-related genes was significantly different in high risk group and low risk group. Among them, the expression levels of *ACSL3*, *ALOX12*, *CRYAB*, *LPCAT3*, *PTGS2*, *SLC1A5*, and *ZEB1* were higher in the high risk group, while the levels of HSBP1 and SLC7A11 were lower in the high risk group. We further verified the above results in TCGA test set and ICGC cohort. The results showed that the risk scores of patients also had close positive correlations with eosinophils, iDC, macrophages, neutrophils, NK CD56dim cells, NK cells, Tem, and Tgd, while risk scores had negative correlations with NK CD56 bright cells, pDC, and TFH (Figures S2(a)–S2(d)). ICGC results also showed that the risk scores and the nine ferroptosis-related genes had strong correlations with most of the immune cells, such as eosinophils, Th2 cells, Tgd cells, cytotoxic cells, pDC, and Th1 cells (Figure S3(a)–S3(d)). Above all, we can summarize that the risk score and the nine ferroptosis-related genes were associated with multiple immune cells.

### 3.7. Functional Analysis

**Figure 7:**
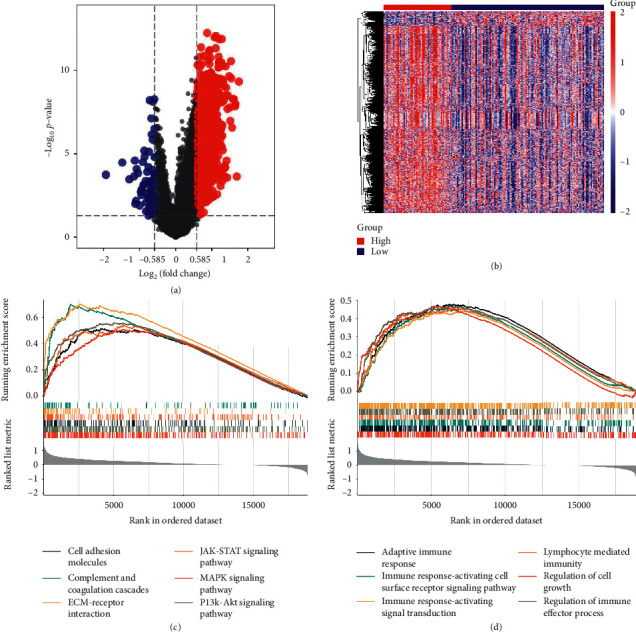
Functional analysis of DEGs between high risk group and low risk group. (a) Volcano map of DEGs. (b) Heatmap of DEGs. (c) KEGG analysis shows significant signaling pathways. (d) GO analysis shows significant GO terms.

Gene Set Enrichment Analysis (GSEA) was conducted to find the key pathways and biological functions that differentiate the different groups. First, the volcano map and heatmap between two groups are drawn in Figures 7(a) and 7(b). Then, KEGG analysis and GO analysis were conducted and the results showed that the DEGs were mainly enriched in cell adhesion molecules, complement and coagulation cascades, ECM-receptor interaction, JAK-STAT signaling pathway, MAPK signaling pathway, PI3K-Akt signaling pathway, and so on, which were not only iron-related but also immune-related. Interestingly, DEGs between high risk group and low risk group also were enriched in several immune-related GO terms such as adaptive immune response, immune response-activating cell surface receptor signaling pathway, immune response-activating signal transduction, lymphocyte mediated immunity, regulation of cell growth, regulation of immune effector process, and so on, suggesting that the signature may be involved in these pathways and thus influence the survival of OC.

### 3.8. Combining Riskscore with Clinical Characteristics

**Figure 8:**
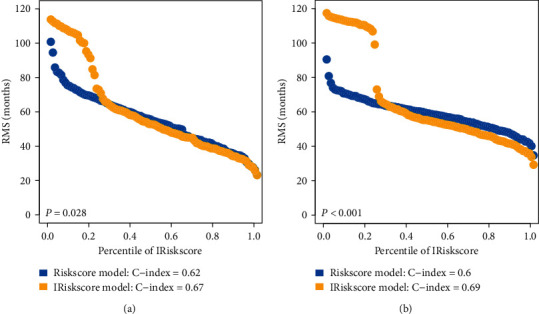
RMS curves of nine ferroptosis-related genes' signature for patients in TCGA training set (a) and TCGA test set (b).

In addition to Riskscore, we also affirmed that clinical characteristics (i.e., tumor status) served as independent prognostic factors, which could have complementary values (Table 2). To further improve the prognostic accuracy, we combined Riskscore with the major clinical variables using the coefficients generated from multivariate cox regression analysis in the TCGA training cohort and generated a new integrative model IRiskscore as follows: IRiskscore = 5.578 × Riskscore + 2.240 × tumor status. However, due to the lack of tumor status information in ICGC cohort, the integrated model of IRiskscore was further applied to the TCGA training cohort and test cohort where full clinical information was available. Significant improvement in estimation of restricted mean survival (RMS) was achieved with the continuous form of IRiskscore relative to Riskscore (C-index: 0.67 vs 0.62 in the TCGA training cohort, *p* < 0.05; C-index: 0.69 vs 0.6 in TCGA test cohort, *p* < 0.001; Figures 8(a) and 8(b)).

## 4. Discussion

OC is still a challenging disease to human beings, especially women, with high incidence and morbidity. In recent years, large efforts have been made to unveil the etiology and mechanism in order to expand the landscape of OC therapeutic [22, 23]. Selective induction of cancer cell death is the most effective therapy method of malignant tumor [24]. Increasing evidence showed that ferroptosis plays a vital role in tumorigenesis and cancer therapeutics [10, 17]. However, the number of ferroptosis-related researches in OC is still very small and the systematic analysis of OC has yet to be elucidated. In the present study, we first constructed a prognostic model integrating nine ferroptosis-related genes in TCGA training set, including *LPCAT3*, *ACSL3*, *CRYAB*, *PTGS2*, *ALOX12*, *HSBP1*, *SLC1A5*, *SLC7A11*, and *ZEB1*. Then, the constructed model was validated in TCGA test set and ICGC cohort. Further, using the ssGSEA method, we estimated the abundance of immune cell populations and found that the risk scores and the nine ferroptosis genes had strong correlations with most of immune cells, such as eosinophils, iDC, macrophages, neutrophils, NK cells, Tem, Tgd, and Th1 cells, suggesting strong connections between the nine ferroptosis-related genes and immune status. Finally, we also integrated with other clinical factors and we found the synthesized clinical factors and ferroptosis-related gene signature improved prognostic accuracy relative to the ferroptosis-related gene signature alone.

In this study, the constructed prognostic model was composed of nine ferroptosis-related genes and they were reported to be involved in the development of several diseases. *LPCAT3*, an enzyme that converts lysophosphatidylcholine to phosphatidylcholine in the liver, could maintain the systemic homeostasis and participate in the phospholipid remodeling and intestinal stem cell growth and tumorigenesis [25, 26]. *ACSL3*, an androgen-responsive gene involved in the generation of fatty acyl-CoA esters, could promote intratumoral steroidogenesis in prostate cancer cells [27]. *CRYAB*, a member of the small heat shock protein family, could regulate several signaling pathways including PI3K/AKT and ERK pathways in cancers [28, 29]. *PTGS2*, also named cyclooxygenase-2, targeting the PGE2/NF-kappaB pathway, could promote the proliferation and serve as an anti-inflammatory drug target in OC [30, 31]. *ALOX12*, a member of a nonheme lipoxygenase family of dioxygenases, plays a crucial role in ALOX12-12HETE-GPR31 signaling axis and was dysregulated in recurrence of hepatocellular carcinoma [32]. *ALOX12* is also required for p53-mediated tumor suppression through a distinct ferroptosis pathway [33]. Lin28A could enrich HSBP1 and upregulate its expression and then regulate the stem-like properties of OC [34]. *SLC1A5* protects patients with non-serous OC from recurrent disease, presumably by means of biological mechanisms that are unrelated to cytotoxic drug sensitivity [35]. The SLC7A11-encoded cystine transporter supplies cells with cysteine which is a key source of GSH [36]. Antisense lncRNA As-SLC7A11 suppresses epithelial ovarian cancer progression mainly by targeting SLC7A11 [37]. *ZEB1*, best known for driving an epithelial-to-mesenchymal transition (EMT) in cancer cells to promote tumor progression, is required by tumor-associated macrophages (TAMs) for their tumor-promoting and chemotherapy resistance functions in a mouse model of ovarian cancer [38].

Based on the prognostic model, we divided patients into high risk groups and low risk groups from TCGA and ICGC cohort and then risk scores were calculated using the formula. After validation of the prognostic model, ssGSEA method was used for identifying the relationship between ferroptosis and tumor immunity. Interestingly, the immune cells and these nine genes were significant between high risk groups and low risk groups. Although the mechanisms of OC still remain largely unknown, the research we performed took an insight into several pathways in OC based on the concept of ferroptosis and immune status. Based on functional analysis, KEGG pathway enrichment analysis showed the DEGs were mainly enriched in cell adhesion molecules [39], JAK-STAT signaling pathway [40], MAPK signaling pathway [41], PI3K-Akt signaling pathway [42], ECM-receptor interaction [43], complement and coagulation cascades [44, 45], focal adhesion [46–48], and so on, which were not only iron-related but also immune-related. Interestingly, DEGs between high risk group and low risk group were found enriched in several immune-related GO terms such as adaptive immune response [49], immune response-activating cell surface receptor signaling pathway [50], immune response-activating signal transduction, lymphocyte mediated immunity, regulation of cell growth, regulation of immune effector process, and so on.

To our knowledge, this ferroptosis-related gene signature has not been previously reported and it will provide assistance to clinical practice. First, in this model, we only need targeted sequencing based on specific genes which greatly reduces the financial burden of patients. Second, it also does not require the identification of somatic mutations and copy number variation in patients. Third, we can detect the expression of these genes by single cell sequencing in circulating tumor cells to patients who are poor candidates for surgery. In addition, we also integrated with other clinical factors and we found the synthesized clinical factors and ferroptosis-related gene signature improved prognostic accuracy relative to the ferroptosis-related gene signature alone, which may become routinely used in the future.

However, there are still several limits of our present study. First, all data processed in this study were publicity data. The real world data need to be warranted to verify our results. Second, although we have tested the robustness of our model several times, the intrinsic weakness is still inevitable. Finally, experimental studies need to be carried out to investigate the functional roles and confirm the presence of gene products by immunohistochemistry of the nine genes in OC in future work.

In summary, this study constructed a model containing 9 ferroptosis-related genes. The model was validated to be associated with OS in the TCGA training set, TCGA test set, and ICGC cohort. The ssGSEA method demonstrated that ferroptosis had a tight link with tumor immunity but needs further experimental validation.

## 5. Conclusions

This is the first study to report a novel ferroptosis-related prognostic model to predict OS of OC.

## Figures and Tables

**Table 1:** Clinical characteristics of OC patients in TCGA.

Characteristic	TCGA
*Age*	
Median	58
Range	38–83

*Race*	
White	326
Black or African American	25
Asian	12
American Indian or Alaska Native	2
Native Hawaiian or other Pacific Islander	1
NA	11

*Clinical stage*	
Stage I	1
Stage II	21
Stage III	292
Stage IV	57
NA	6

*Tumor grade*	
G1	1
G2	44
G3	320
G4	3
GX	6
NA	3

*Tumor status*	
Tumor-free	71
With tumor	263
NA	43

*Vital status*	
Alive	145
Dead	232

**Table 2:** Univariable and multivariable cox regression analysis of the nine ferroptosis-related gene signature and survival analysis of OC patients in the TCGA training, test set, and ICGC cohort.

Variables			The training set (*n* = 189)				The test set (*n* = 188)				The ICGC set (*n* = 93)			
			HR	95% CI of HR		*p*	HR	95% CI of HR		*p*	HR	95% CI of HR		*p*
				Lower	Upper			Lower	Upper			Lower	Upper	
Univariate analysis	Age	≥60 vs <60	1.166	0.905	1.501	0.235	1.156	0.883	1.513	0.292	1.169	0.842	1.622	0.352
	Race	White vs others	0.715	0.449	1.138	0.157	0.665	0.444	0.995	0.047	—	—	—	—
	Stage	III, IV vs I, II	1.758	0.781	3.958	0.173	1.682	0.746	3.797	0.21	0.686	0.408	1.154	0.156
	Grade	G3, G4 vs G1, G2	1.074	0.732	1.577	0.715	1.279	0.835	1.959	0.258	—	—	—	—
	Tumor status	Tumor vs tumor-free	5.067	2.244	11.441	<0.001	7.222	2.654	19.655	<0.001	—	—	—	—
	Risk score	High vs low	2.657	1.823	3.872	<0.001	1.887	1.287	2.768	0.001	3.115	1.914	5.069	<0.001

Multivariable analysis	Age	≥60 vs <60	1.09	0.822	1.443	0.55	1.201	0.897	1.607	0.218	1.193	0.857	1.66	0.297
	Race	White vs others	0.707	0.429	1.165	0.174	0.668	0.437	1.02	0.062	—	—	—	—
	Stage	III, IV vs I, II	1.742	0.729	4.163	0.211	1.292	0.474	3.523	0.616	0.79	0.466	1.338	0.38
	Grade	G3, G4 vs G2, G1	0.986	0.63	1.541	0.949	1.115	0.69	1.803	0.657	—	—	—	—
	Tumor status	Tumor vs tumor-free	4.58	2.017	10.401	<0.001	7.672	2.803	20.996	<0.001	—	—	—	—
	Risk score	High vs low	1.767	1.155	2.704	0.009	1.944	1.271	2.973	0.002	3.06	1.865	5.021	<0.001

## Data Availability

The data used to support the results are available at the TCGA (https://tcga-data.nci.nih.gov/tcga/) and ICGC (https://dcc.icgc.org/).

## Abbreviations

HR: Hazard ratio
CI: Confidence interval.

## Supplementary Materials

Supplementary Materials Figure S1: Kaplan–Meier estimates of the overall survival of patients with different clinical factors (age, tumor status, stage, and grade) in training set, test set, and ICGC set. Figure S2: Comparison of the ssGSEA scores between different risk groups in the TCGA test set. (a) The risk score between the nine ferroptosis-related genes and different immune cells. (b) Heatmap of the different groups and components. (c) Detailed risk scores and comparison in high risk group and low risk group. (d) The expression level and comparison of nine ferroptosis-related genes in high risk group and low risk group. The meaning of the statistical difference is as follows: ^*∗*^*p* < 0.05, ^*∗∗*^*p* < 0.01, and ^*∗∗∗*^*p* < 0.001. Figure S3. Comparison of the ssGSEA scores between different risk groups in the ICGC cohort. (a) The risk score between the nine ferroptosis-related genes and different immune cells. (b) Heatmap of the different groups and components. (c) Detailed risk scores and comparison in high risk group and low risk group. (d) The expression level and comparison of nine ferroptosis-related genes in high risk group and low risk group. The meaning of the statistical difference is as follows: ^*∗*^*p* < 0.05, ^*∗∗*^*p* < 0.01, and ^*∗∗∗*^*p* < 0.001. Table S1: ferroptosis-related genes. Table S2: ferroptosis-related genes associated with OS.

## References

[1] Bray F., Ferlay J., Soerjomataram I., Siegel R. L., Torre L. A., Jemal A. (2018). Global cancer statistics 2018: GLOBOCAN estimates of incidence and mortality worldwide for 36 cancers in 185 countries. *CA: A Cancer Journal for Clinicians*.

[2] Torre L. A., Trabert B., DeSantis C. E. (2018). Ovarian cancer statistics, 2018. *CA: A Cancer Journal for Clinicians*.

[3] Lheureux S., Braunstein M., Oza A. M. (2019). Epithelial ovarian cancer: evolution of management in the era of precision medicine. *CA: A Cancer Journal for Clinicians*.

[4] Rooth C. (2013). Ovarian cancer: risk factors, treatment and management. *British Journal of Nursing*.

[5] Chandra A., Pius C., Nabeel M. (2019). Ovarian cancer: current status and strategies for improving therapeutic outcomes. *Cancer Medicine*.

[6] Penninkilampi R., Eslick G. D. (2018). Perineal talc use and ovarian cancer. *Epidemiology*.

[7] Cho K. R., Shih I.-M. (2009). Ovarian cancer. *Annual Review of Pathology: Mechanisms of Disease*.

[8] Hirschhorn T., Stockwell B. R. (2019). The development of the concept of ferroptosis. *Free Radical Biology and Medicine*.

[9] Dixon S. J. (2017). Ferroptosis: bug or feature?. *Immunological Reviews*.

[10] Liang C., Zhang X., Yang M., Dong X. (2019). Recent progress in ferroptosis inducers for cancer therapy. *Advanced Materials*.

[11] Tesfay L., Paul B. T., Konstorum A. (2019). Stearoyl-CoA desaturase 1 protects ovarian cancer cells from ferroptotic cell death. *Cancer Research*.

[12] Carbone M., Melino G. (2019). Stearoyl CoA desaturase regulates ferroptosis in ovarian cancer offering new therapeutic perspectives. *Cancer Research*.

[13] Yang W.-H., Huang Z., Wu J., Ding C.-K. C., Murphy S. K., Chi J.-T. (2020). A TAZ-ANGPTL4-NOX2 axis regulates ferroptotic cell death and chemoresistance in epithelial ovarian cancer. *Molecular Cancer Research*.

[14] Yang W. S., SriRamaratnam R., Welsch M. E. (2014). Regulation of ferroptotic cancer cell death by GPX4. *Cell*.

[15] Liu H., Schreiber S. L., Stockwell B. R. (2018). Targeting dependency on the GPX4 lipid peroxide repair pathway for cancer therapy. *Biochemistry*.

[16] Stockwell B. R., Friedmann Angeli J. P., Bayir H. (2017). Ferroptosis: a regulated cell death nexus linking metabolism, redox biology, and disease. *Cell*.

[17] Hassannia B., Vandenabeele P., Vanden Berghe T. (2019). Targeting ferroptosis to iron out cancer. *Cancer Cell*.

[18] Bersuker K., Hendricks J. M., Li Z. (2019). The CoQ oxidoreductase FSP1 acts parallel to GPX4 to inhibit ferroptosis. *Nature*.

[19] Doll S., Freitas F. P., Shah R. (2019). FSP1 is a glutathione-independent ferroptosis suppressor. *Nature*.

[20] Heagerty P. J., Lumley T., Pepe M. S. (2000). Time-dependent ROC curves for censored survival data and a diagnostic marker. *Biometrics*.

[21] Bindea G., Mlecnik B., Tosolini M. (2013). Spatiotemporal dynamics of intratumoral immune cells reveal the immune landscape in human cancer. *Immunity*.

[22] Eisenhauer E. A. (2017). Real-world evidence in the treatment of ovarian cancer. *Annals of Oncology*.

[23] Ottevanger P. B. (2017). Ovarian cancer stem cells more questions than answers. *Seminars in Cancer Biology*.

[24] Liu H.-j., Hu H.-m., Li G.-z. (2020). Ferroptosis-related gene signature predicts glioma cell death and glioma patient progression. *Frontiers in Cell and Developmental Biology*.

[25] Cheng C.-W., Yilmaz Ö. H. (2018). FAOund the link: phospholipid remodeling and intestinal stem cell growth and tumorigenesis. *Cell Stem Cell*.

[26] Wang B., Tontonoz P. (2019). Phospholipid remodeling in physiology and disease. *Annual Review of Physiology*.

[27] Migita T., Takayama K. i., Urano T. (2017). ACSL3 promotes intratumoral steroidogenesis in prostate cancer cells. *Cancer Science*.

[28] Zhang J., Liu J., Wu J., Li W., Chen Z., Yang L. (2019). Progression of the role of CRYAB in signaling pathways and cancers. *OncoTargets and Therapy*.

[29] Ruan H., Li Y., Wang X. (2020). CRYAB inhibits migration and invasion of bladder cancer cells through the PI3K/AKT and ERK pathways. *Japanese Journal of Clinical Oncology*.

[30] Barnard M. E., Hecht J. L., Rice M. S. (2018). Anti-inflammatory drug use and ovarian cancer risk by COX1/COX2 expression and infiltration of tumor-associated macrophages. *Cancer Epidemiology Biomarkers & Prevention*.

[31] Zhang X., Yan K., Deng L. (2019). Cyclooxygenase 2 promotes proliferation and invasion in ovarian cancer cells via the PGE2/NF-*κ*B pathway. *Cell Transplantation*.

[32] Yang F., Zhang Y., Ren H. (2019). Ischemia reperfusion injury promotes recurrence of hepatocellular carcinoma in fatty liver via ALOX12-12HETE-GPR31 signaling axis. *Journal of Experimental & Clinical Cancer Research*.

[33] Chu B., Kon N., Chen D. (2019). ALOX12 is required for p53-mediated tumour suppression through a distinct ferroptosis pathway. *Nature Cell Biology*.

[34] Zhong Y., Cao L., Ma H. (2020). Lin28A regulates stem-like properties of ovarian cancer cells by enriching RAN and HSBP1 mRNA and up-regulating its protein expression. *International Journal of Biological Sciences*.

[35] Bjersand K., Seidal T., Sundström-Poromaa I., Åkerud H., Skirnisdottir I. (2017). The clinical and prognostic correlation of HRNPM and SLC1A5 in pathogenesis and prognosis in epithelial ovarian cancer. *PLoS One*.

[36] Ogiwara H., Takahashi K., Sasaki M. (2019). Targeting the vulnerability of glutathione metabolism in ARID1A-deficient cancers. *Cancer Cell*.

[37] Yuan J., Liu Z., Song R. (2017). Antisense lncRNA As-SLC7A11 suppresses epithelial ovarian cancer progression mainly by targeting SLC7A11. *Die Pharmazie*.

[38] Cortés M., Sanchez‐Moral L., de Barrios O. (2017). Tumor‐associated macrophages (TAMs) depend on ZEB1 for their cancer‐promoting roles. *The EMBO Journal*.

[39] Figliuolo da Paz V. R., Figueiredo-Vanzan D., Dos Santos Pyrrho A. (2019). Interaction and involvement of cellular adhesion molecules in the pathogenesis of Schistosomiasis mansoni. *Immunology Letters*.

[40] Owen K. L., Brockwell N. K., Parker B. S. (2019). JAK-STAT signaling: a double-edged sword of immune regulation and cancer progression. *Cancers*.

[41] Jakhar R., Sharma C., Paul S., Kang S. C. (2018). Immunosuppressive potential of astemizole against LPS activated T cell proliferation and cytokine secretion in RAW macrophages, zebrafish larvae and mouse splenocytes by modulating MAPK signaling pathway. *International Immunopharmacology*.

[42] Vergadi E., Ieronymaki E., Lyroni K., Vaporidi K., Tsatsanis C. (2017). Akt signaling pathway in macrophage activation and M1/M2 polarization. *The Journal of Immunology*.

[43] Mardpour S., Hamidieh A. A., Taleahmad S., Sharifzad F., Taghikhani A., Baharvand H. (2019). Interaction between mesenchymal stromal cell‐derived extracellular vesicles and immune cells by distinct protein content. *Journal of Cellular Physiology*.

[44] De Luca C., Colangelo A. M., Alberghina L., Papa M. (2018). Neuro-immune hemostasis: homeostasis and diseases in the central nervous system. *Frontiers in Cellular Neuroscience*.

[45] Min L., Cheng J., Zhao S. (2016). Plasma-based proteomics reveals immune response, complement and coagulation cascades pathway shifts in heat-stressed lactating dairy cows. *Journal of Proteomics*.

[46] Soenen S. J. H., Nuytten N., De Meyer S. F., De Smedt S. C., De Cuyper M. (2010). High intracellular iron oxide nanoparticle concentrations affect cellular cytoskeleton and focal adhesion kinase-mediated signaling. *Small*.

[47] Ehnert S., Linnemann C., Aspera-Werz R. (2018). Immune cell induced migration of osteoprogenitor cells is mediated by TGF-*β* dependent upregulation of NOX4 and activation of focal adhesion kinase. *International Journal of Molecular Sciences*.

[48] Osipov A., Saung M. T., Zheng L., Murphy A. G. (2019). Small molecule immunomodulation: the tumor microenvironment and overcoming immune escape. *Journal for ImmunoTherapy of Cancer*.

[49] Netea M. G., Schlitzer A., Placek K., Joosten L. A. B., Schultze J. L. (2019). Innate and adaptive immune memory: an evolutionary continuum in the host’s response to pathogens. *Cell Host & Microbe*.

[50] Ke Z. B., Wu Y. P., Huang P. (2020). Identification of novel genes in testicular cancer microenvironment based on ESTIMATE algorithm‐derived immune scores. *Journal of Cellular Physiology*.

