# Supplementary material for: Ferroptosis-Related Gene Model to Predict Overall Survival of Ovarian Carcinoma
Source: J Oncol. 2021 Jan 13;2021:6687391. doi: 10.1155/2021/6687391 (PMC7817275; doi:10.1155/2021/6687391)

**Supplementary material:**

**Ferroptosis-related Gene Model to Predict Overall Survival of Ovarian Carcinoma**

Liuqing Yang^1^, Saisai Tian^2^, Yun Chen^1^, Chenyun Miao^3^, Ying Zhao^3^, Ruye Wang^3^, Qin Zhang^1^*

^1^Guangxing Hospital Affiliated to Zhejiang Chinese Medical University, Hangzhou, Zhejiang, 310007, P.R. China.

^2^Department of Phytochemistry, School of Pharmacy, the Second Military Medical University, Shanghai 200433, P. R. China.

^3^Zhejiang Chinese Medical University, Hangzhou, Zhejiang, 310053, P. R. China.

*Correspondence: Qin Zhang, zhaqin01@163.com; Department of TCM Gynecology, Guangxing Hospital Affiliated to Zhejiang Chinese Medical University, No. 453 Tiyuchang Road, Hangzhou, Zhejiang 310007, P. R. China; Tel.: +86 13606528580

**Supplementary Tables**

**Table S1 Ferrotosis-related genes.**

| **Table S1 Ferrotosis-related genes** | |
| --- | --- |
| Ferrotosis-related genes | Name |
| ACSL4 | acyl-CoA synthetase long-chain family member 4 |
| AKR1C1 | aldo-keto reductase family 1 member C1 |
| AKR1C2 | aldo-keto reductase family 1 member C2 |
| AKR1C3 | aldo-keto reductase family 1 member C3 |
| ALOX15 | arachidonate 15-lipoxygenase |
| ALOX5 | arachidonate 5-lipoxygenase |
| ALOX12 | arachidonate 12-lipoxygenase |
| ATP5MC3 | ATP synthase membrane subunit c locus 3 |
| CARS | cysteinyl tRNA synthetase |
| CBS | cystathion ine beta synthase |
| CD44 | CD44 molecule |
| CHAC1 | ChaC glutathione- specific gamma-glutamyl cyclotransferase 1 |
| CISD1 | CDGSH iron sulfur domain 1 |
| CS | citrate synthase |
| DPP4 | dipeptidyl-dippeptidase-4 |
| FANCD2 | Fanconi anemia comple mentation group D2 |
| GCLC | glutamate-cysteine ligase catalytic subunit |
| GCLM | glutamate-cysteine ligase modifier subunit |
| GLS2 | glutaminase 2 |
| GPX4 | glutathio ne peroxidase 4 |
| GSS | glutathione synthetase |
| HMGCR | 3-hydroxy-3- methylglutaryl-CoA reductase |
| HSPB1 | heat shock protein beta 1 |
| CRYAB | heat shock protein beta 5 |
| LPCAT3 | lysophosp hatidylcholine acyltransferase 3 |
| MT1G | metallothionein-1G |
| NCOA4 | nuclear receptor coactiva tor 4 |
| PTGS2 | prostagla ndin-endoperoxide synthase 2 |
| RPL8 | ribosomal protein L8 |
| SAT1 | spermidine/spermine N1-acetyltra nsferase 1 |
| SLC7A11 | solute carrier family 7 member 11 |
| FDFT1 | farnesyl-diphosphate farnesyltransferase 1 |
| TFRC | transferrin receptor |
| TP53 | tumor protein 53 |
| EMC2 | ER membrane protein complex subunit 2 |
| AIFM2 | apoptosis inducing factor mitochondria associated 2 |
| PHKG2 | phospho rylase kinase ,g2 |
| HSBP1 | heat-shock 27-k Da protein 1 |
| ACO1 | aconitase 1 |
| FTH1 | ferritin heavy chain 1 |
| STEAP3 | six-transm embrane epithelial antigen of prostate 3 |
| NFS1 | cysteine desulfurase |
| ACSL3 | acyl-CoA synthetase long-chain family member 3 |
| ACACA | Acetyl-CoA carboxylase alpha |
| PEBP1 | phosphatidy lethanolamine-binding protein 1 |
| ZEB1 | zinc finger E-box-binding homeobox 1 |
| SQLE | squalene monooxygenase |
| FADS2 | fatty acid desaturase 2/acyl-CoA 6-desaturase |
| NFE2L2 | nuclear factor, erythroid 2 like 2 |
| KEAP1 | kelch-like ECH- associated protein 1 |
| NQO1 | quinone oxidoreductas e-1 |
| NOX1 | NADPH oxidase 1 |
| ABCC1 | ATP binding cassette subfamily C member 1 |
| SLC1A5 | solute carrier family 1 member 5 |
| GOT1 | glutamic-oxa loacetic transaminase 1 |
| G6PD | glucose-6-phosphate dehydrogenas e |
| PGD | phosphoglycerate dehydrogenas e |
| IREB2 | iron response element-binding protein 2 |
| HMOX1 | heme oxygenase 1 |
| ACSF2 | acyl-CoA synthetase family member 2 |

**Table S2 Ferroptosis-related genes associated with OS**

| **Table S2 Ferroptosis-related genes associated with OS** | | | | | |
| --- | --- | --- | --- | --- | --- |
| Gene | Beta | HR | 95% CI of HR (lower) | 95% CI of HR (upper) | P.value |
| ACSL4 | 0.327 | 1.386 | 1.111 | 1.730 | 0.004 |
| ZEB1 | 0.237 | 1.267 | 1.039 | 1.546 | 0.019 |
| LPCAT3 | 0.248 | 1.281 | 1.011 | 1.622 | 0.040 |
| ACSF2 | 0.197 | 1.218 | 0.996 | 1.490 | 0.055 |
| SLC1A5 | 0.199 | 1.220 | 0.970 | 1.534 | 0.090 |
| GOT1 | 0.251 | 1.286 | 0.959 | 1.724 | 0.093 |
| CRYAB | 0.087 | 1.091 | 0.983 | 1.209 | 0.100 |
| ACSL3 | 0.290 | 1.337 | 0.943 | 1.893 | 0.103 |
| HMGCR | 0.204 | 1.226 | 0.960 | 1.567 | 0.103 |
| PTGS2 | 0.144 | 1.154 | 0.970 | 1.373 | 0.106 |
| ALOX12 | 0.194 | 1.214 | 0.957 | 1.540 | 0.110 |
| SLC7A11 | -0.134 | 0.875 | 0.738 | 1.037 | 0.123 |
| STEAP3 | 0.117 | 1.124 | 0.965 | 1.308 | 0.134 |
| PGD | -0.190 | 0.827 | 0.642 | 1.065 | 0.142 |
| HSBP1 | -0.275 | 0.759 | 0.526 | 1.097 | 0.143 |

**Supplementary Figures**

**Figure S1 Kaplan-Meier estimates of the overall survival of patients with different clinical factors (age, tumor status, stage and grade).**

**
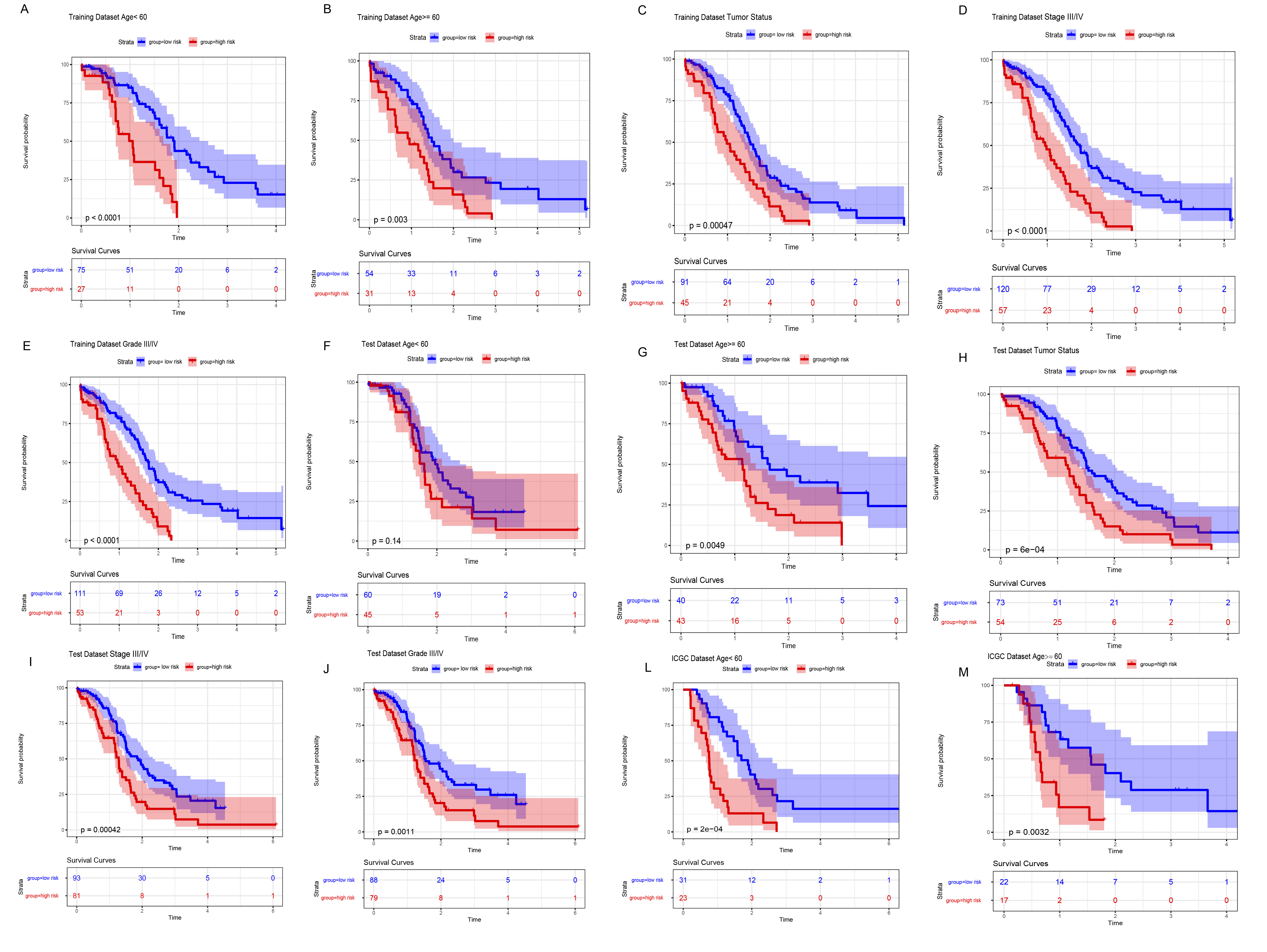
**

**Figure S2** Comparison of the ssGSEA scores between different risk groups in the TCGA test set (A) The correlation between the nine ferroptosis related genes and risk score and different immune cells (B) Heatmap of the immune cell infiltration between different groups (C) detailed risk scores and comparison in high risk group and low risk group (D) the expression level and comparison of nine ferroptosis related genes in high risk group and low risk group. The meaning of the statistical difference is as follows: * represents P < 0.05, ** represents P < 0.01 and *** represents P < 0.001.





**Figure S3** Comparison of the ssGSEA scores between different risk groups in the ICGC cohort (A) The correlation between the nine ferroptosis related genes and risk score and different immune cells (B) Heatmap of the immune cell infiltration between different groups (C) detailed risk scores and comparison in high risk group and low risk group (D) the expression level and comparison of nine ferroptosis related genes in high risk group and low risk group. The meaning of the statistical difference is as follows: * represents P < 0.05, ** represents P < 0.01 and *** represents P < 0.001.


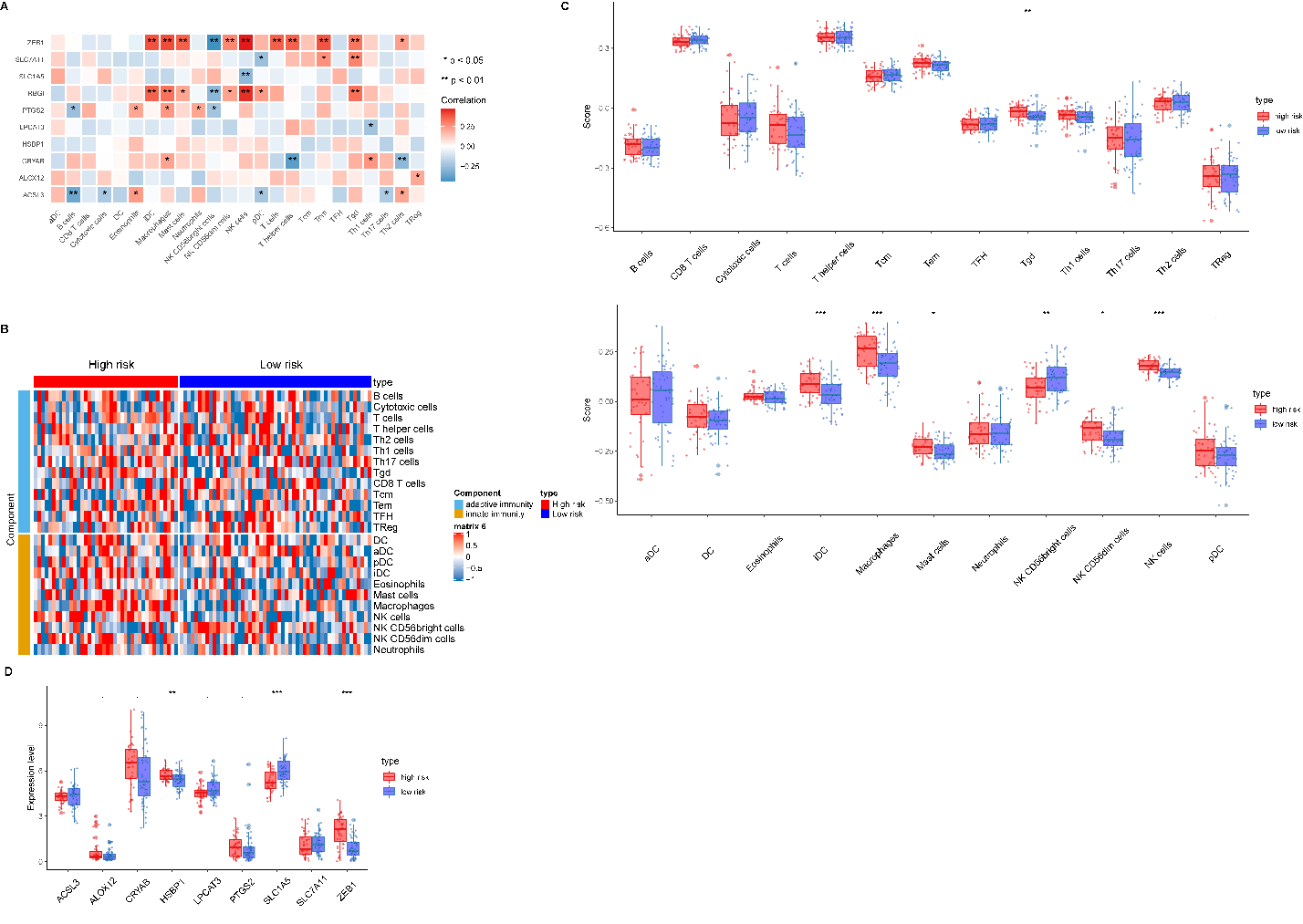

Supplement: Supplementary Materials — Figure S1: Kaplan–Meier estimates of the overall survival of patients with different clinical factors (age, tumor status, stage, and grade) in training set, test set, and ICGC set. Figure S2: Comparison of the ssGSEA scores between different risk groups in the TCGA test set. (a) The risk score between the nine ferroptosis-related genes and different immune cells. (b) Heatmap of the different groups and components. (c) Detailed risk scores and comparison in high risk group and low risk group. (d) The expression level and comparison of nine ferroptosis-related genes in high risk group and low risk group. The meaning of the statistical difference is as follows: ∗p < 0.05, ∗∗p < 0.01, and ∗∗∗p < 0.001. Figure S3. Comparison of the ssGSEA scores between different risk groups in the ICGC cohort. (a) The risk score between the nine ferroptosis-related genes and different immune cells. (b) Heatmap of the different groups and components. (c) Detailed risk scores and comparison in high risk group and low risk group. (d) The expression level and comparison of nine ferroptosis-related genes in high risk group and low risk group. The meaning of the statistical difference is as follows: ∗p < 0.05, ∗∗p < 0.01, and ∗∗∗p < 0.001. Table S1: ferroptosis-related genes. Table S2: ferroptosis-related genes associated with OS. [file 6687391.f1.zip › 6687391.f1/Supplementary material (2).docx]
