# Supplementary figures and images for: Ferroptosis-Related Gene Model to Predict Overall Survival of Ovarian Carcinoma
Source: J Oncol. 2021 Jan 13;2021:6687391. doi: 10.1155/2021/6687391 (PMC7817275; doi:10.1155/2021/6687391)

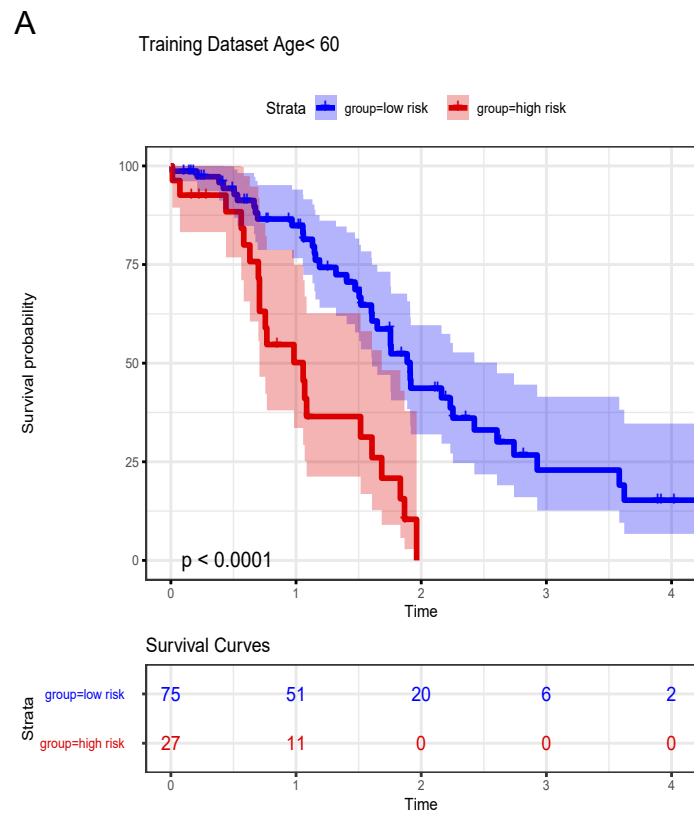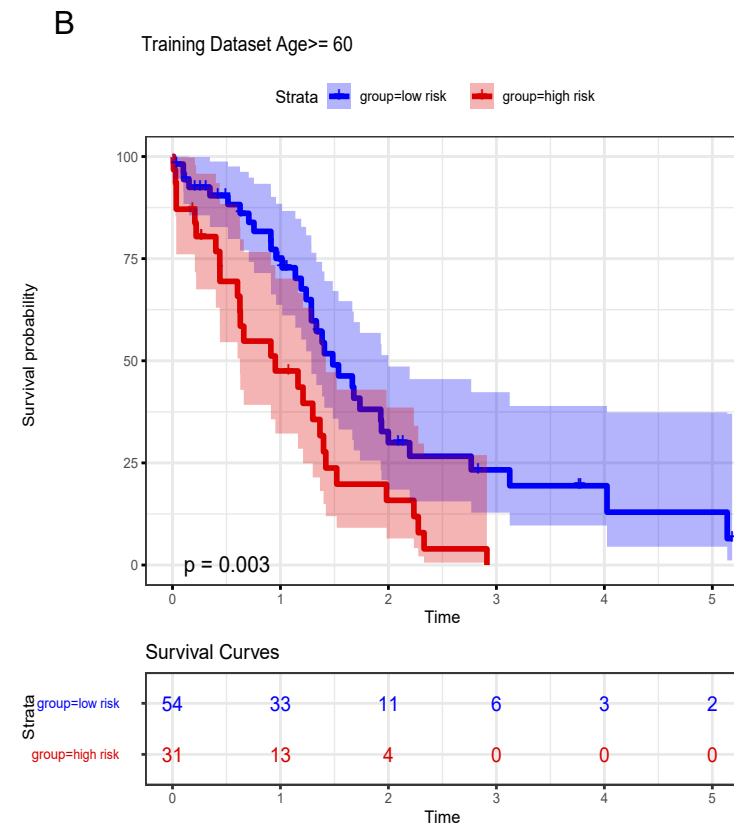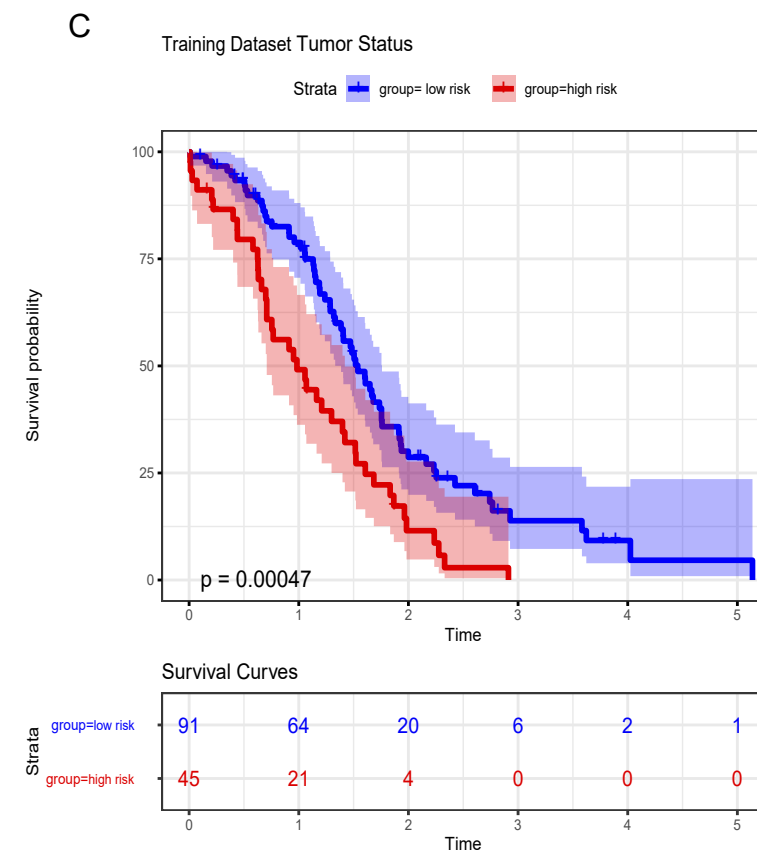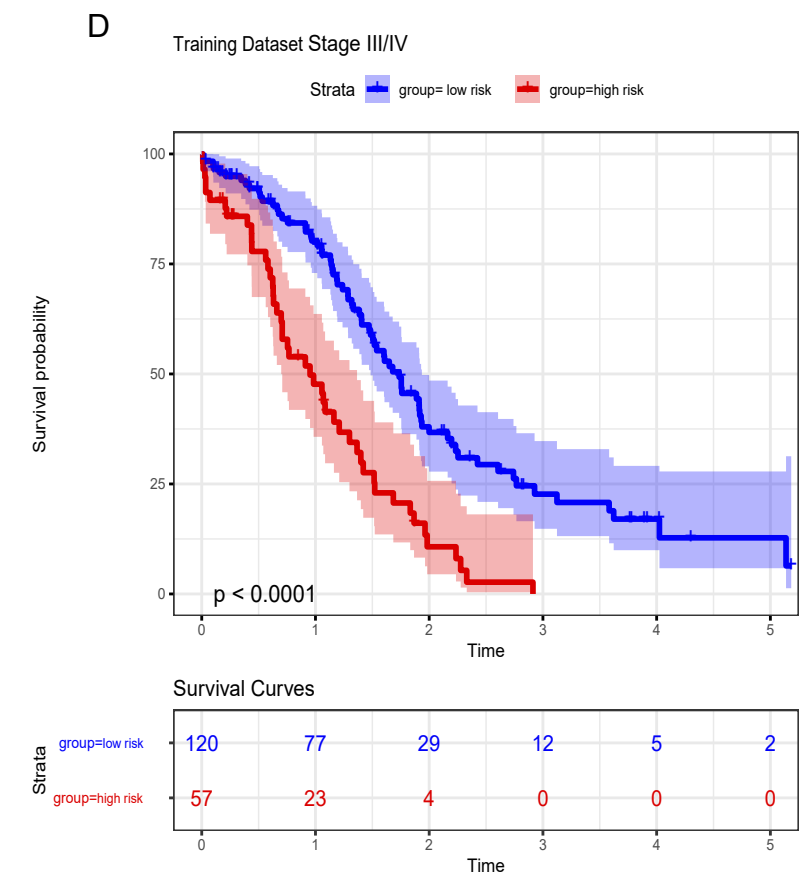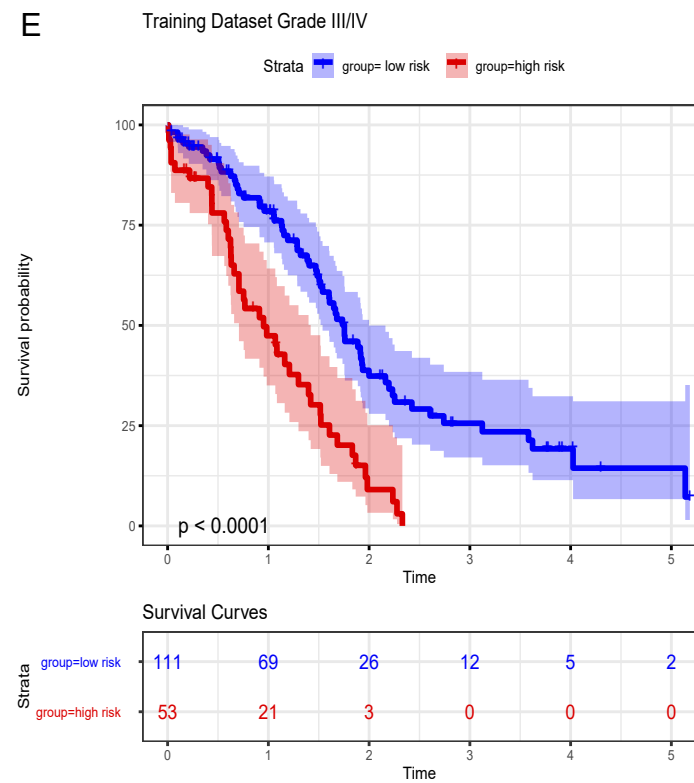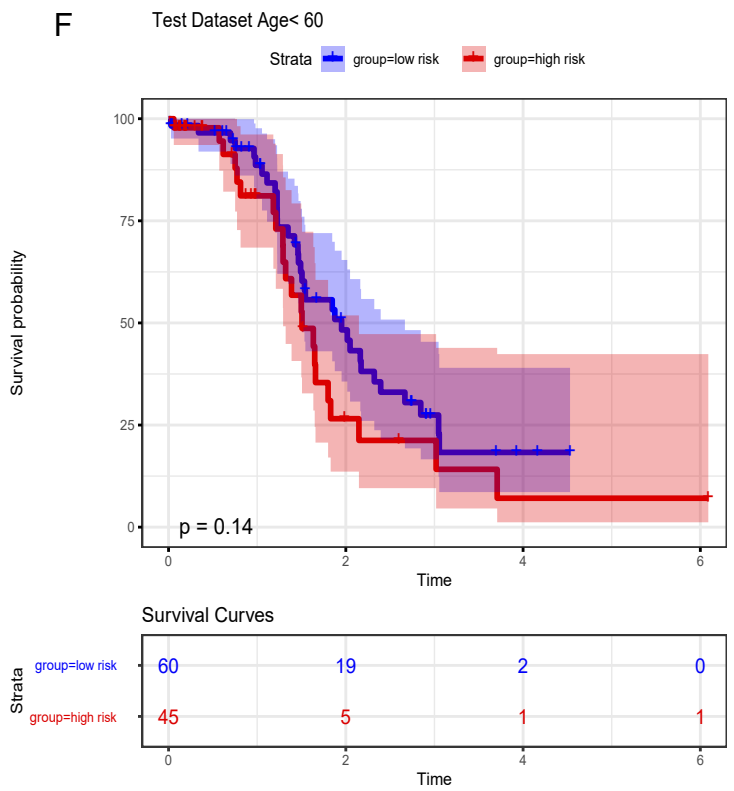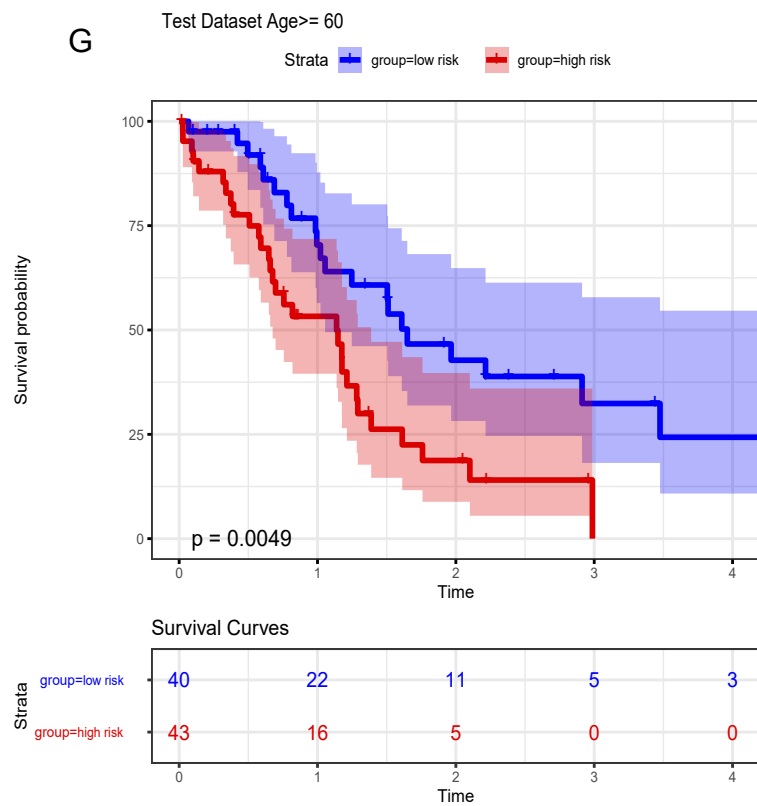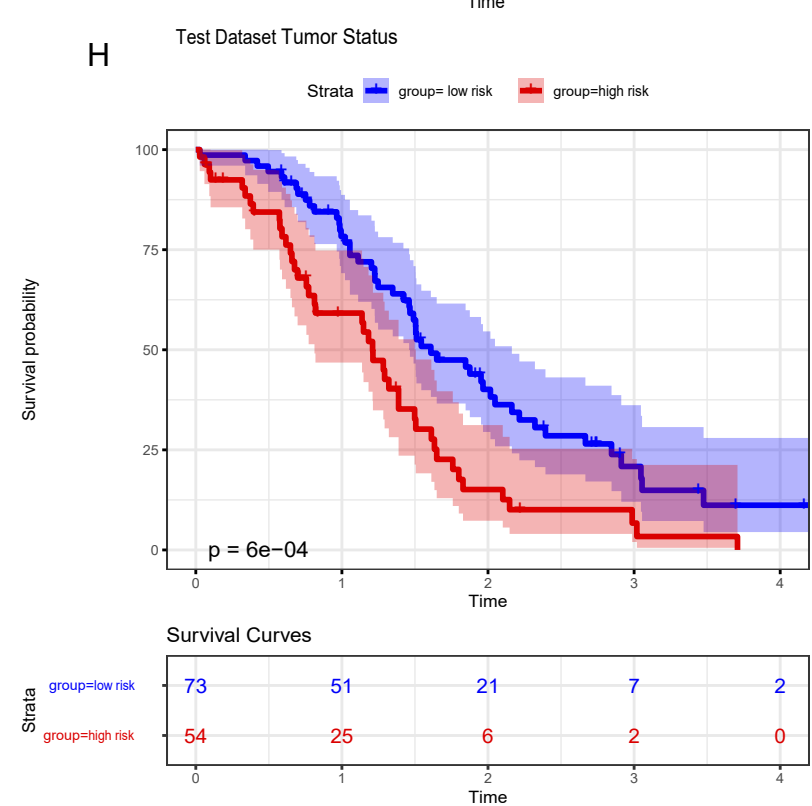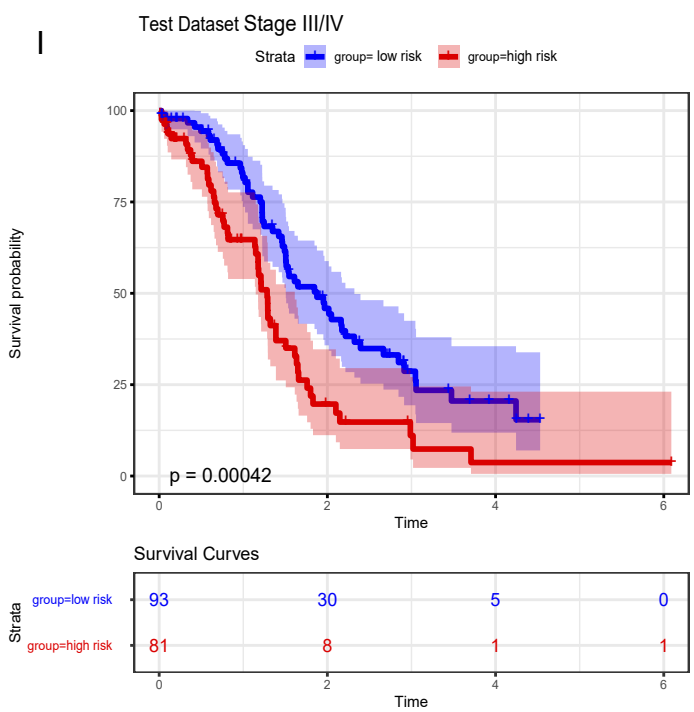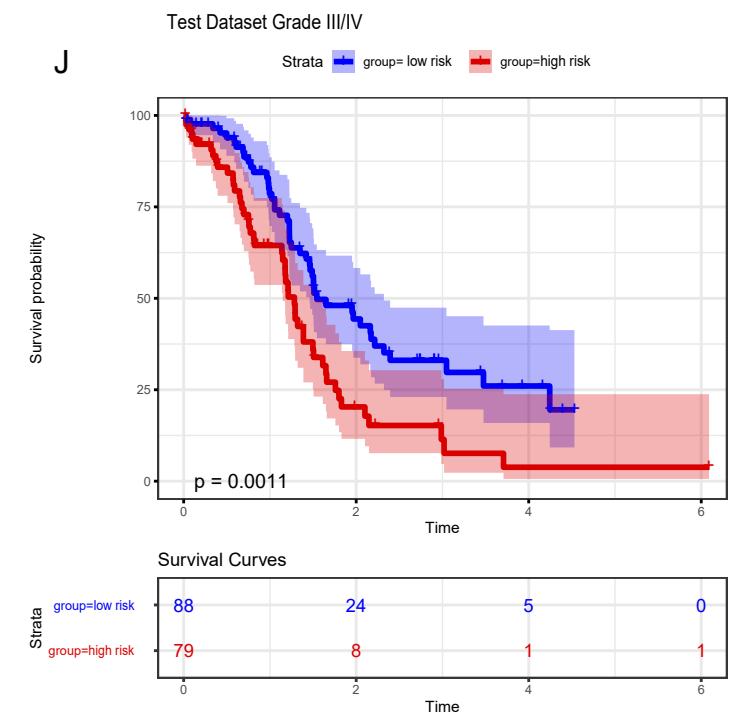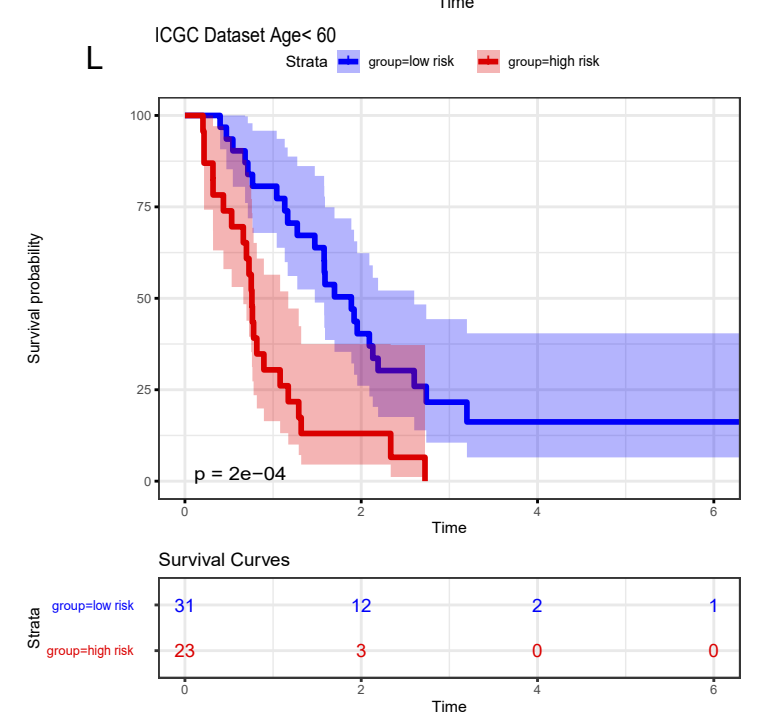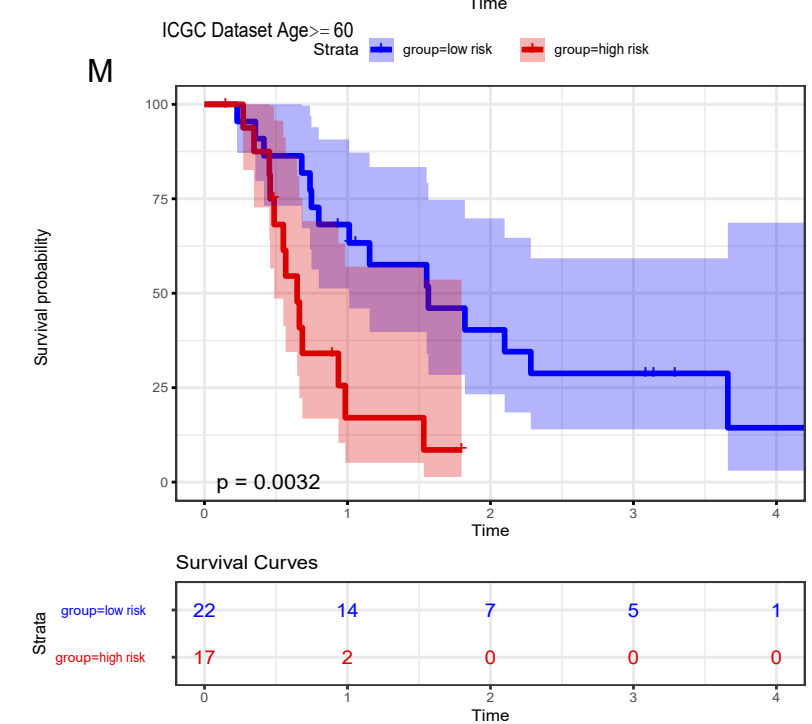

Supplement: Supplementary Materials — Figure S1: Kaplan–Meier estimates of the overall survival of patients with different clinical factors (age, tumor status, stage, and grade) in training set, test set, and ICGC set. Figure S2: Comparison of the ssGSEA scores between different risk groups in the TCGA test set. (a) The risk score between the nine ferroptosis-related genes and different immune cells. (b) Heatmap of the different groups and components. (c) Detailed risk scores and comparison in high risk group and low risk group. (d) The expression level and comparison of nine ferroptosis-related genes in high risk group and low risk group. The meaning of the statistical difference is as follows: ∗p < 0.05, ∗∗p < 0.01, and ∗∗∗p < 0.001. Figure S3. Comparison of the ssGSEA scores between different risk groups in the ICGC cohort. (a) The risk score between the nine ferroptosis-related genes and different immune cells. (b) Heatmap of the different groups and components. (c) Detailed risk scores and comparison in high risk group and low risk group. (d) The expression level and comparison of nine ferroptosis-related genes in high risk group and low risk group. The meaning of the statistical difference is as follows: ∗p < 0.05, ∗∗p < 0.01, and ∗∗∗p < 0.001. Table S1: ferroptosis-related genes. Table S2: ferroptosis-related genes associated with OS. [file 6687391.f1.zip › 6687391.f1/figure S1.pdf]

**A**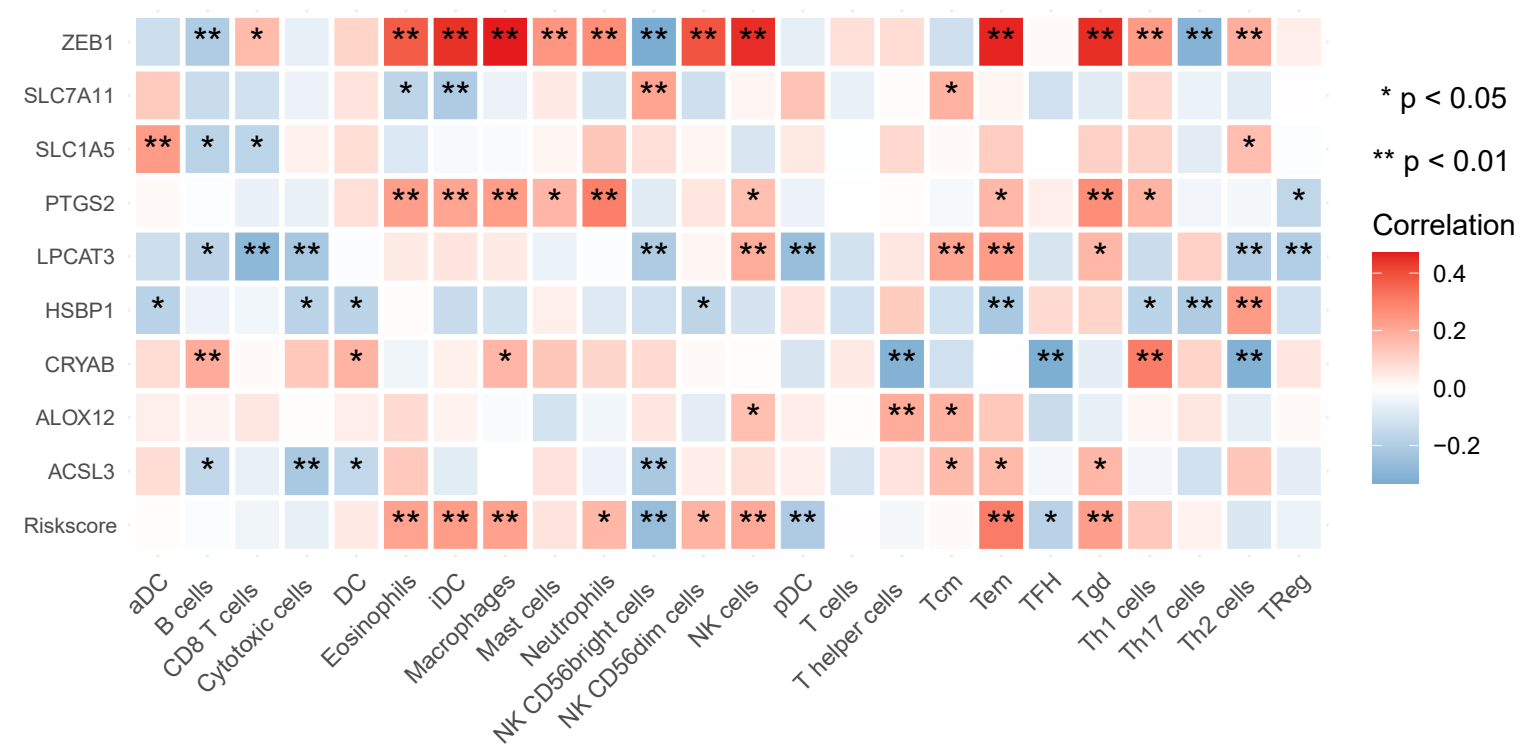**B**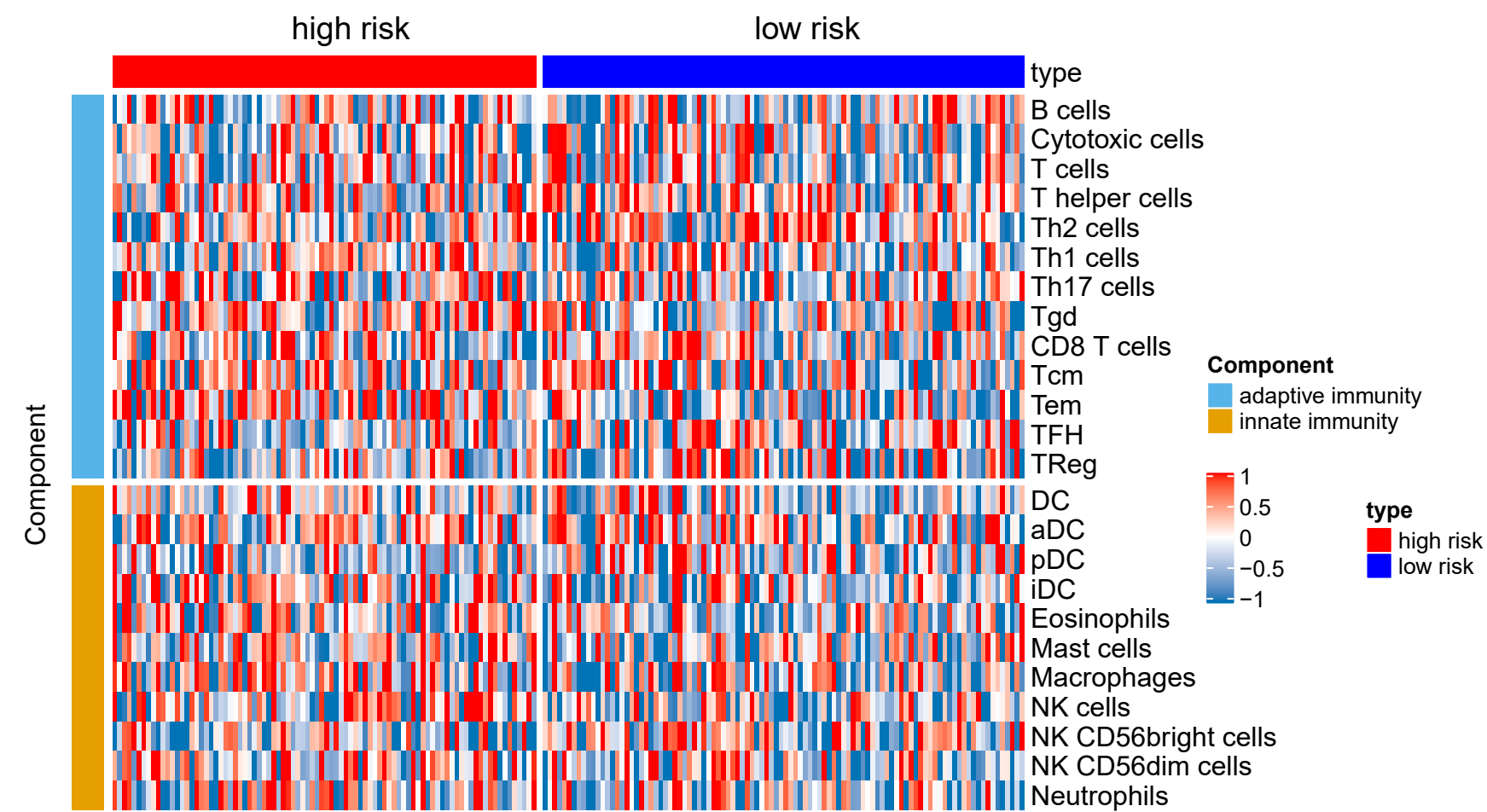**D**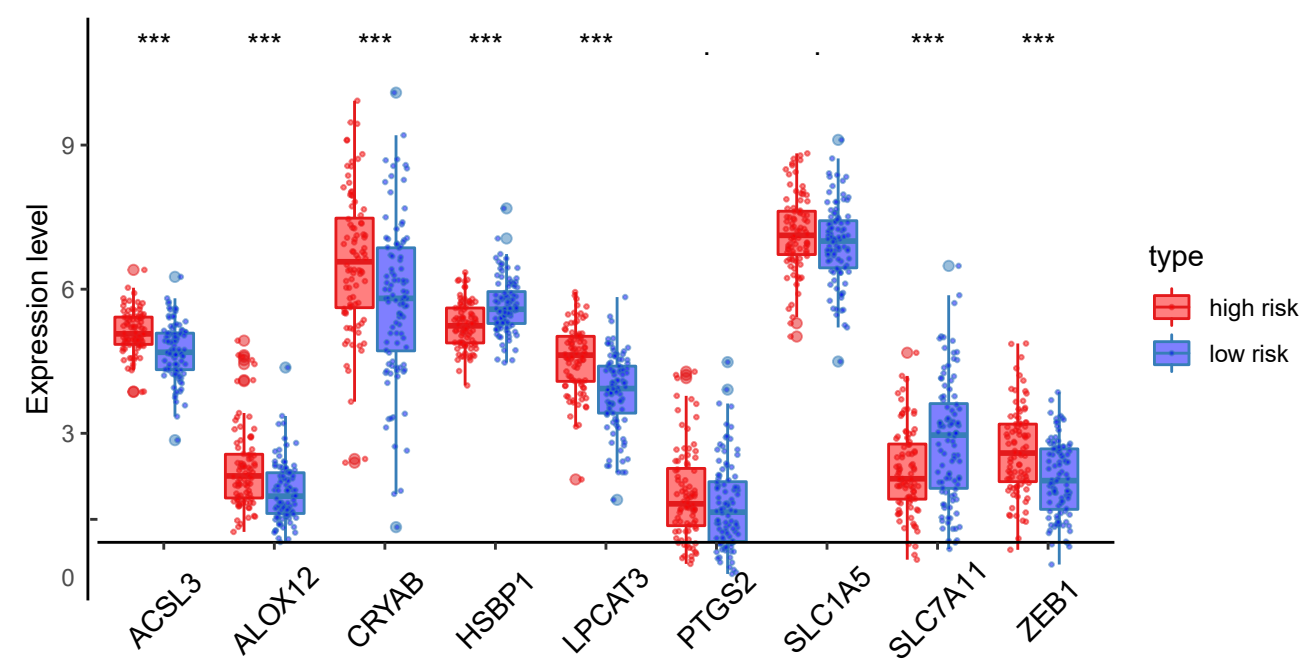**C**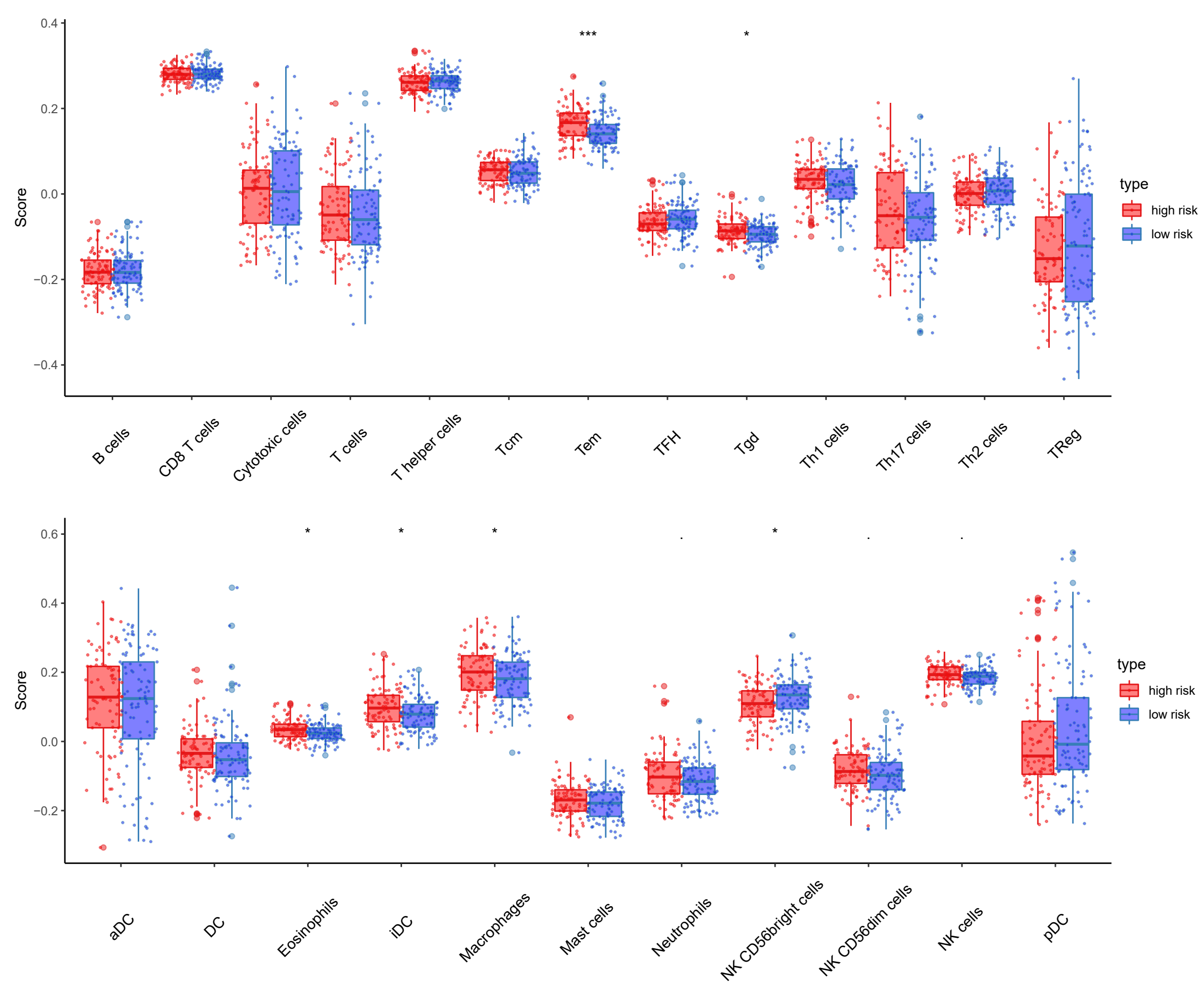

Supplement: Supplementary Materials — Figure S1: Kaplan–Meier estimates of the overall survival of patients with different clinical factors (age, tumor status, stage, and grade) in training set, test set, and ICGC set. Figure S2: Comparison of the ssGSEA scores between different risk groups in the TCGA test set. (a) The risk score between the nine ferroptosis-related genes and different immune cells. (b) Heatmap of the different groups and components. (c) Detailed risk scores and comparison in high risk group and low risk group. (d) The expression level and comparison of nine ferroptosis-related genes in high risk group and low risk group. The meaning of the statistical difference is as follows: ∗p < 0.05, ∗∗p < 0.01, and ∗∗∗p < 0.001. Figure S3. Comparison of the ssGSEA scores between different risk groups in the ICGC cohort. (a) The risk score between the nine ferroptosis-related genes and different immune cells. (b) Heatmap of the different groups and components. (c) Detailed risk scores and comparison in high risk group and low risk group. (d) The expression level and comparison of nine ferroptosis-related genes in high risk group and low risk group. The meaning of the statistical difference is as follows: ∗p < 0.05, ∗∗p < 0.01, and ∗∗∗p < 0.001. Table S1: ferroptosis-related genes. Table S2: ferroptosis-related genes associated with OS. [file 6687391.f1.zip › 6687391.f1/figure S2.pdf]

**A**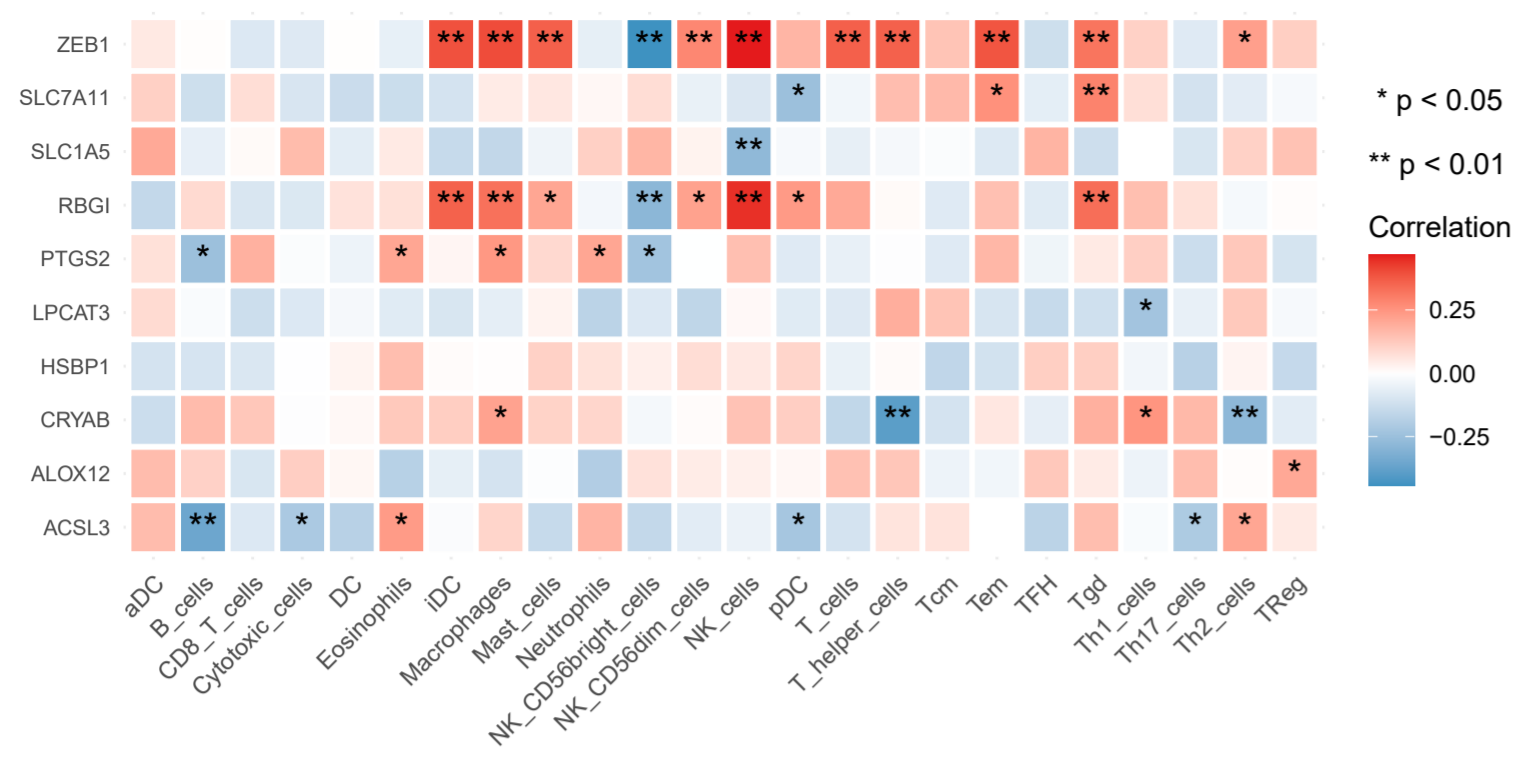**C**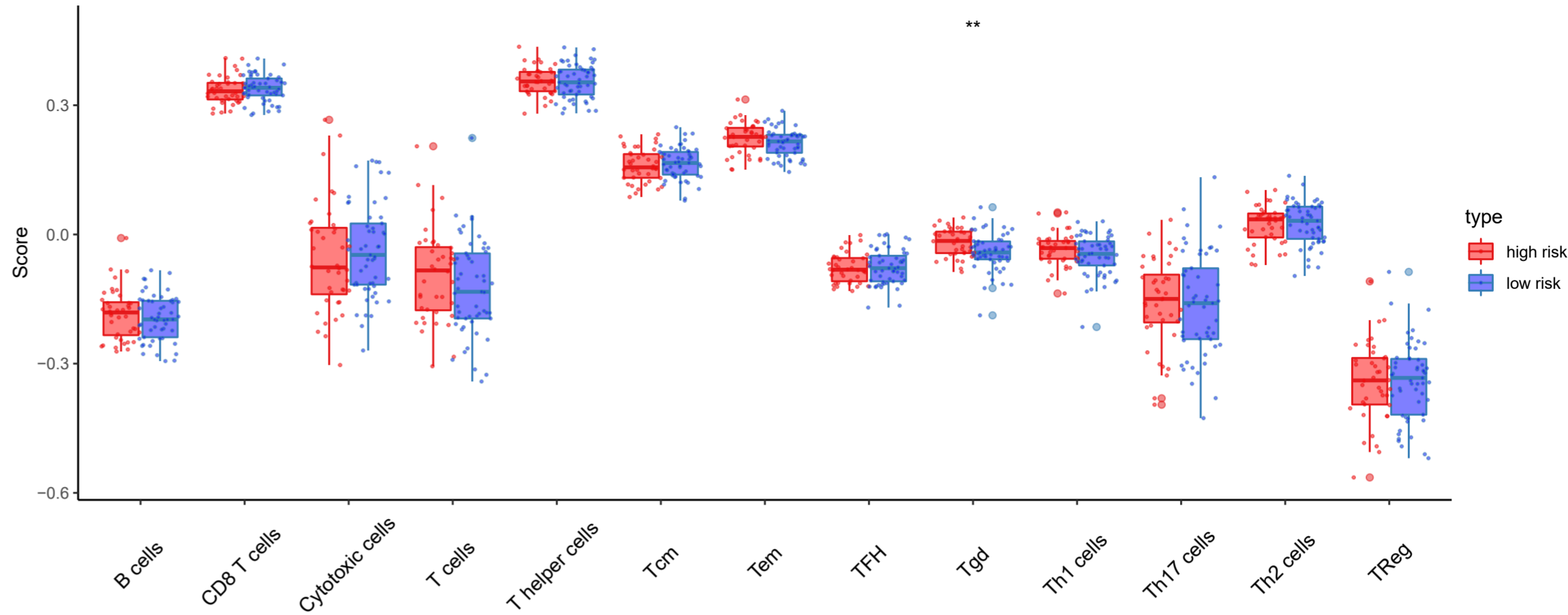**B**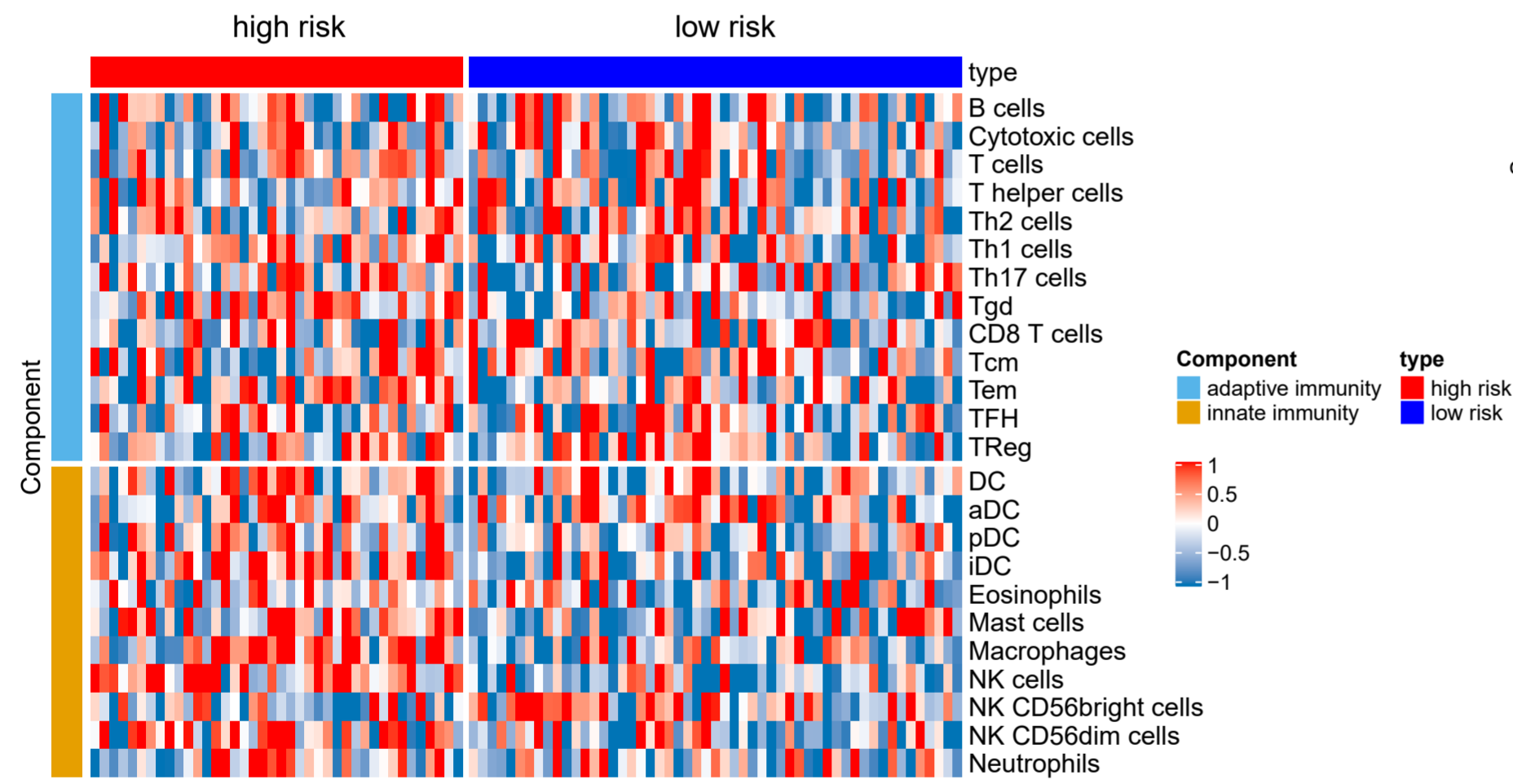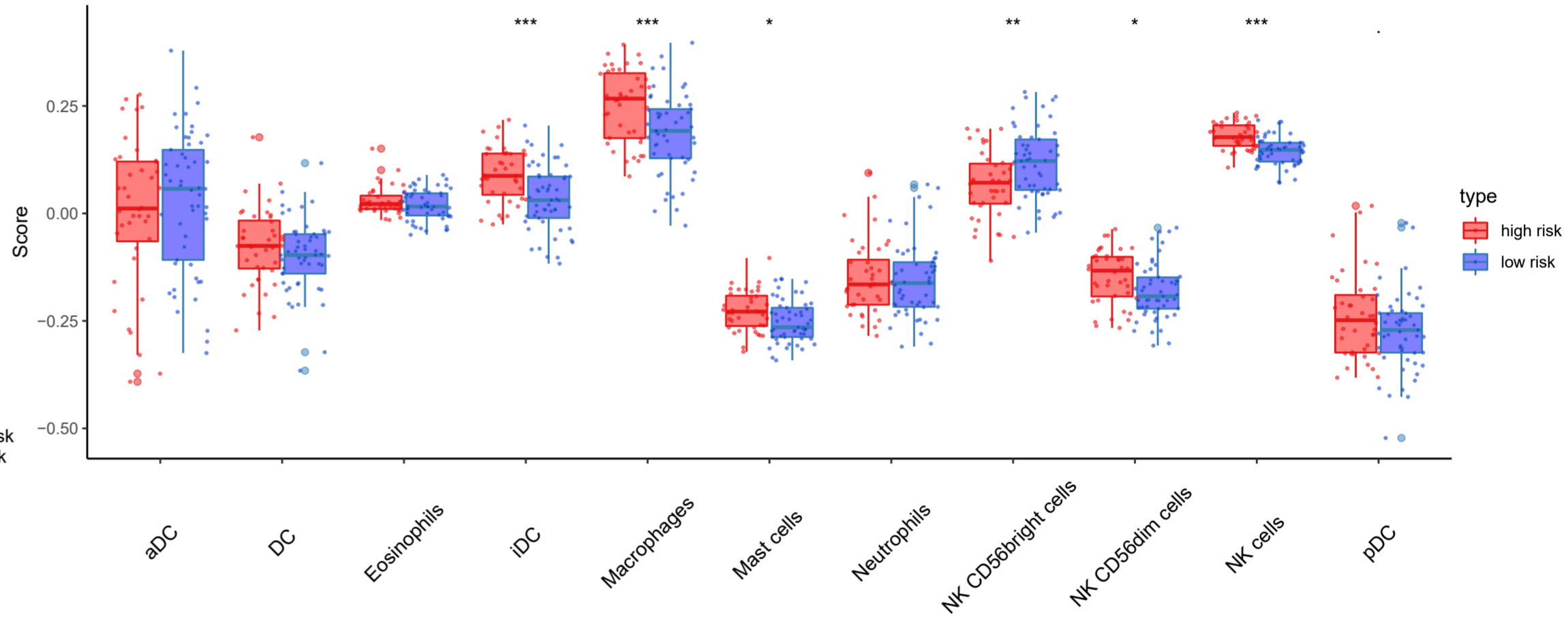**D**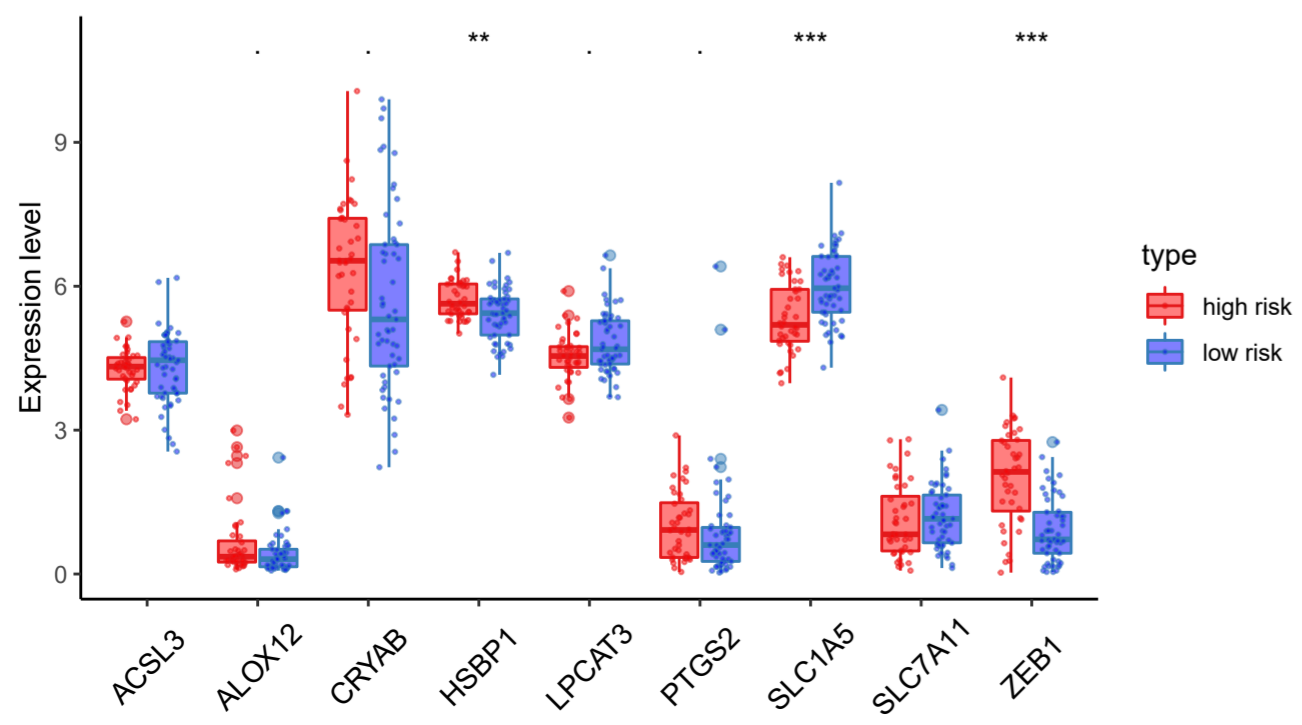

Supplement: Supplementary Materials — Figure S1: Kaplan–Meier estimates of the overall survival of patients with different clinical factors (age, tumor status, stage, and grade) in training set, test set, and ICGC set. Figure S2: Comparison of the ssGSEA scores between different risk groups in the TCGA test set. (a) The risk score between the nine ferroptosis-related genes and different immune cells. (b) Heatmap of the different groups and components. (c) Detailed risk scores and comparison in high risk group and low risk group. (d) The expression level and comparison of nine ferroptosis-related genes in high risk group and low risk group. The meaning of the statistical difference is as follows: ∗p < 0.05, ∗∗p < 0.01, and ∗∗∗p < 0.001. Figure S3. Comparison of the ssGSEA scores between different risk groups in the ICGC cohort. (a) The risk score between the nine ferroptosis-related genes and different immune cells. (b) Heatmap of the different groups and components. (c) Detailed risk scores and comparison in high risk group and low risk group. (d) The expression level and comparison of nine ferroptosis-related genes in high risk group and low risk group. The meaning of the statistical difference is as follows: ∗p < 0.05, ∗∗p < 0.01, and ∗∗∗p < 0.001. Table S1: ferroptosis-related genes. Table S2: ferroptosis-related genes associated with OS. [file 6687391.f1.zip › 6687391.f1/figure S3.pdf]
